# An Overview of 24 Years of Molecular Phylogenetic Studies in *Phallales* (*Basidiomycota*) With Notes on Systematics, Geographic Distribution, Lifestyle, and Edibility

**DOI:** 10.3389/fmicb.2021.689374

**Published:** 2021-07-09

**Authors:** Gislaine C. S. Melanda, Alexandre G. S. Silva-Filho, Alexandre Rafael Lenz, Nelson Menolli, Alexandro de Andrade de Lima, Renato Juciano Ferreira, Nathalia Mendonça de Assis, Tiara S. Cabral, María P. Martín, Iuri Goulart Baseia

**Affiliations:** ^1^Centro de Biociências, Departamento de Micologia, Programa de Pós-Graduação em Biologia de Fungos, Universidade Federal de Pernambuco (UFPE), Recife, Brazil; ^2^Centro de Biociências, Departamento de Botânica e Zoologia, Programa de Pós-Graduação em Sistemática e Evolução, Universidade Federal do Rio Grande do Norte (UFRN), Natal, Brazil; ^3^Departamento de Ciências Exatas e da Terra, Colegiado de Sistemas de Informação, Universidade do Estado da Bahia (UNEB), Salvador, Brazil; ^4^Departamento de Ciências e Matemática, Subárea de Biologia, Instituto Federal de Educação, Ciência e Tecnologia de São Paulo (IFSP), São Paulo, Brazil; ^5^Núcleo de Pesquisa em Micologia, Instituto de Botânica (IBt), São Paulo, Brazil; ^6^Departamento de Ciências Biológicas, Universidade Regional do Cariri (URCA), Crato, Brazil; ^7^Programa de Pós-Graduação em Genética, Conservação e Biologia Evolutiva, Instituto Nacional de Pesquisas da Amazônia (INPA), Manaus, Brazil; ^8^Departamento de Micología, Real Jardín Botánico – CSIC, Madrid, Spain

**Keywords:** gasteroid fungi, GenBank, phylogeny, stinkhorns, UNITE

## Abstract

The order *Phallales* (*Basidiomycota*) is represented by gasteroid fungi with expanded and sequestrate basidiomata, known as stinkhorns and false truffles. In phalloids, the first DNA sequence was published in 1997, and after that, some studies aimed to resolve phylogenetic conflicts and propose new species based on DNA markers; however, the number of families and genera in the order still generates controversies among researchers. Thus, this work aims to provide an overview of *Phallales* diversity represented by selected DNA markers available in public databases. We retrieved *Phallales* sequences from DNA databases (GenBank and UNITE) of seven markers: ITS (internal transcribed spacer), nuc-LSU (nuclear large subunit rDNA), nuc-SSU (nuclear small subunit rDNA), mt-SSU (mitochondrial small subunit rDNA), *ATP*6 (ATPase subunit 6), *RPB*2 (nuclear protein-coding second largest subunit of RNA polymerase), and *TEF*1-α (translation elongation factor subunit 1α). To compose our final dataset, all ITS sequences retrieved were subjected to BLASTn searches to identify additional ITS sequences not classified as *Phallales*. Phylogenetic analyses based on Bayesian and maximum likelihood approaches using single and combined markers were conducted. All ITS sequences were clustered with a cutoff of 98% in order to maximize the number of species hypotheses. The geographic origin of sequences was retrieved, as well as additional information on species lifestyle and edibility. We obtained a total of 1,149 sequences, representing 664 individuals. Sequences of 41 individuals were unidentified at genus level and were assigned to five distinct families. We recognize seven families and 22 genera in *Phallales*, although the delimitation of some genera must be further revisited in order to recognize only monophyletic groups. Many inconsistencies in species identification are discussed, and the positioning of genera in each family is shown. The clustering revealed 118 species hypotheses, meaning that approximately 20% of all described species in *Phallales* have DNA sequences available. Information related to geographic distribution represents 462 individuals distributed in 46 countries on all continents, except Antarctica. Most genera are saprotrophic with only one putative ectomycorrhizal genus, and 2.1% of the legitimate specific names recognized in *Phallales* are confirmed edible species. Great progress in the molecular analyses of phalloids has already been made over these years, but it is still necessary to solve some taxonomic inconsistencies, mainly at genus level, and generate new data to expand knowledge of the group.

## Introduction

The first molecular analyses including gasteroid fungi (Hibbett et al., 1997) showed that they represent a polyphyletic and artificial grouping of taxa that share a common ancestor with gilled and nongilled mushrooms that have active dispersion of the basidiospores. Hibbett et al. (1997) showed the gasteroid–phalloid fungi to group in a clade with other forms of gasteroid fungi, the *Geastraceae* Corda (earthstars) and *Sphaerobolaceae* J. Schröt. (cannonballs), all sharing a common ancestor with coralloid fungi. After that, Pine et al. (1999) confirmed the phylogenetic relationship between some cantharelloid, clavarioid, and phalloid fungi, naming all of them the gomphoid–phalloid clade, which was later confirmed as monophyletic (Hibbett and Thorn, 2001; Krüger et al., 2001; Binder and Hibbett, 2002; Hibbett and Binder, 2002) and designated as a subclass of *Agaricomycetes*: *Phallomycetidae* K. Hosaka, Castellano and Spatafora (Hosaka et al., 2006).

*Phallales* E. Fisch (*Agaricomycetes*, *Phallomycetidae*) includes representatives of gasteroid fungi with basidiospores that are passively dispersed mainly by insects and commonly named phalloid fungi, alien fungi, stinkhorns, and lattice or cage stinkhorns (Fischer, 1898; Cunningham, 1931; Miller and Miller, 1988; Pegler and Gomez, 1994). The phalloid fungi are mostly saprobic and characterized by hypogeous or epigeous immature basidiomata that are divided into chambers; thick white rhizomorphs are usually present; peridium with two or three layers, one of which is gelatinous; mature basidiomata that are usually epigeous or partially hypogeous, expanded, or sequestrate; pseudostipitate or sessile receptacle; receptacle carrying the green, olive to brown gleba; the usual presence of gelatinous to mucilaginous gleba that may be powdery at maturity, as in *Gastrosporium* Mattir.; and basidiospores mostly ellipsoid and smooth, with only a few genera with ornamentation on the basidiospore wall, as in *Gastrosporium*, *Kjeldsenia* Colgan, Castellano and Bougher, and *Phlebogaster* Fogel (Hosaka et al., 2006; Trierveiler-Pereira et al., 2014a).

Fischer (1898) grouped in *Phallales* the families *Clathraceae* Chevall. and *Phallaceae* Corda, which include specimens with expanded branched and unbranched basidiomata, respectively. *Lysuraceae* Corda was established in the same article as the establishment of *Phallaceae* (Corda, 1842); both families are characterized by basal pseudostipitate basidiomata, but unlike the morphology in *Phallaceae*, the apical part of the basidiomata in *Lysuraceae* is branched. *Lysuraceae* was not accepted by Fischer (1898) as an independent family in *Phallales*, and their species were considered by this author within *Clathraceae*, a view also adopted by a number of subsequent authors such as Cunningham (1944), Dring (1980), Jülich (1981), Miller and Miller (1988), Pegler and Gomez (1994), Hibbett and Thorn (2001), and Kirk et al. (2008). On the other hand, some authors (He et al., 2019; Wijayawardene et al., 2020) and well-known databases (Nilsson et al., 2019; Index Fungorum, 2020; MycoBank., 2020; Schoch et al., 2020) have placed in *Phallaceae* all the species traditionally classified as *Lysuraceae*. However, Hosaka et al. (2006), Degreef et al. (2013), Trierveiler-Pereira et al. (2014a), and Sulzbacher et al. (2016) consider these three families to be independent (Table 1).

**TABLE 1:** Family classification of *Phallales* and related orders according to published molecular studies and taxonomic and molecular databases.

Work	Families accepted in *Phallales*	Families accepted in *Hysterangiales*	Families accepted in *Boletales*
Hosaka et al. (2006), Degreef et al. (2013)	*Clathraceae*, *Claustulaceae* (=*Gelopellaceae*), *Lysuraceae*, *Phallaceae*, *Protophallaceae*, *Trappeaceae*		
Trierveiler-Pereira et al. (2014a)	*Clathraceae*, *Claustulaceae* (=*Gelopellaceae*), *Gastrosporiaceae*, *Lysuraceae*, *Phallaceae*, *Protophallaceae*, *Trappeaceae*		
Sulzbacher et al. (2016)	*Clathraceae*, *Claustulaceae* (=*Gelopellaceae*), *Lysuraceae*, *Phallaceae*, *Protophallaceae*, *Trappeaceae*		
He et al. (2019), Wijayawardene et al. (2020)	*Claustulaceae*, *Gastrosporiaceae*, *Phallaceae* (=*Clathraceae*; *Lysuraceae*)	*Trappeaceae*, *Phallogastraceae* (=*Protophallaceae*)	
Schoch et al. (2020)	*Clathraceae*, *Phallaceae* (=*Lysuraceae*; = *Protophallaceae*)	*Hysterangiaceae* (=*Trappeaceae*)	*Gastrosporiaceae*
Nilsson et al. (2019)	*Clathraceae*, *Claustulaceae*, *Gastrosporiaceae*, *Phallaceae* (=*Lysuraceae*)	*Hysterangiaceae* (=*Trappeaceae*)	
Index Fungorum (2020)	*Claustulaceae* (=*Gelopellaceae*), *Phallaceae* (=*Clathraceae*; = *Lysuraceae*)	*Phallogastraceae* (=*Protophallaceae*), *Trappeaceae*	*Gastrosporiaceae*
MycoBank. (2020)	*Claustulaceae* (=*Gelopellaceae*), *Gastrosporiaceae*, *Phallaceae* (=*Clathraceae*; = *Lysuraceae*), *Protophallaceae*	*Trappeaceae*	

Cunningham (1931) accepted in *Phallales* sequestrate (truffle-like) basidiomata classified in the genus *Claustula* K.M. Curtis and established this genus as a type of the new monogeneric family *Claustulaceae* G. Cunn. As mentioned by Cunningham (1931), this family shares with other phalloid fungi the gelatinous peridium, immature basidiomata divided into chambers, and elliptical smooth basidiospores. Zeller (1939) also studied sequestrate fungi and proposed two new families: *Gelopellaceae* Zeller and *Protophallaceae* Zeller. However, these two families, as well as *Hysterangiaceae* E. Fisch., have been consolidated within the order *Hysterangiales* K. Hosaka and Castellano, according to Zeller (1939). *Hysterangiales* was first proposed by Zeller (1939), but it was considered a *nomen nudum* because it was published without a description or diagnosis; it was later formally established in Hosaka et al. (2006).

Moreover, Hosaka et al. (2006) established the subclass *Phallomycetidae*, including the new order *Hysterangiales* mentioned previously and the new *Geastrales* K. Hosaka and Castellano. Thus, in the study by Hosaka et al. (2006), *Phallomycetidae* comprises *Geastrales*, *Gomphalles* Jülich., *Hysterangiales*, and *Phallales*, a classification that is also accepted in He et al. (2019). In this way, Hosaka et al. (2006) agreed with Cunningham (1931) in the definition of *Phallales* including families with expanded basidiomata (*Clathraceae*, *Lysuraceae*, *Phallaceae*), as well as with sequestrate ones (*Claustulaceae*, *Trappeaceae* P.M. Kirk, and *Protophallaceae*). Although *Trappeaceae* was provisionally proposed in *Phallales* by Hosaka et al. (2006) with the genera *Phallobata* G. Cunn. and *Trappea* Castellano, the family was formally proposed two years later by Kirk et al. (2008), who classified it as part of *Hysterangiales*. Later, Sulzbacher et al. (2016) proposed a new genus in *Trappeaceae* (*Restingomyces* Sulzbacher, T. Grebenc and Baseia) and also considered the family in *Phallales* as previously pointed out by Hosaka et al. (2006).

After these 24 years of molecular studies, the sampling of some genera and families within molecular phylogenies of *Phallales* is incomplete. For example, *Gastrosporium*, which is part of the monogeneric family *Gastrosporiaceae* Pilát, was first incorporated in a molecular phylogenetic work by Hibbett and Binder (2002), who considered it as a sequestrate member of *Phallales*. This was later confirmed by Trierveiler-Pereira et al. (2014a), He et al. (2019), Kasuya et al. (2020), and Wijayawardene et al. (2020). However, Hosaka et al. (2006) did not include *Gastrosporium* in their phylogeny because of the lack of a protein code gene in their dataset. In the study by Trierveiler-Pereira et al. (2014a), *Trappeaceae* representatives were not included, and they recognized seven families in *Phallales*, which include *Gastrosporiaceae* plus the six families recognized by Hosaka et al. (2006).

**FIGURE 1:**
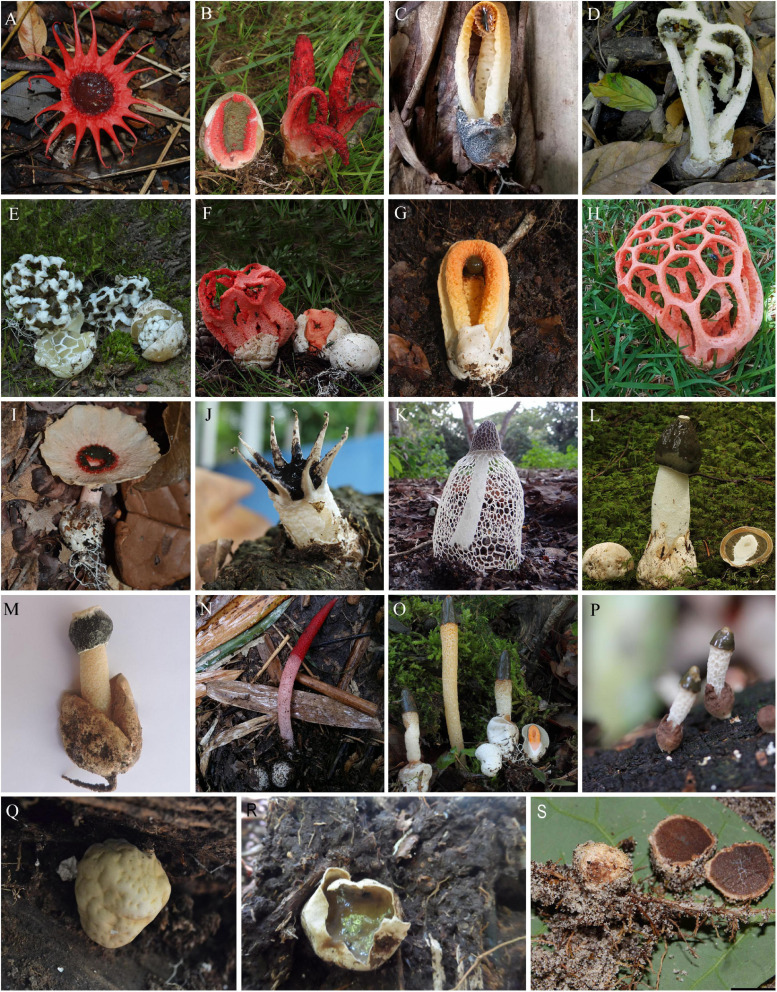
Basidiomata of *Phallales*. *Clathraceae*: **(A)** *Aseroë rubra*; **(B)** *Clathrus archeri* MT13072101B (personal herbarium); **(C)** *Laternea dringii* (coll. AAL94); **(D)** *Blumenavia baturitensis* UFRN-Fungos 2868, holotype (coll. GCSM15); **(E)** *Ileodictyon gracile* MT141003001B (personal herbarium); **(F)** *Clathrus ruber* MT15100406B (personal herbarium); **(G)** *Clathrus columnatus* UFRN-Fungos 2912 (coll. AAL42); **(H)** *Clathrus natalensis* (coll. GSCM30); **(I)** *Abrachium floriforme* UFRN-Fungos 3271. *Lysuraceae*: **(J)** *Lysurus arachnoideus* INPA-Fungos 256537 (coll. TSC41). *Phallaceae*: **(K)** *Phallus indusiatus* INPA-Fungos 264931, neotype (coll. TSC148); **(L)** *Phallus impudicus*; **(M)** *Itajahya* sp. UFRN-Fungos 3342 (coll. AAL106); **(N)** *Mutinus bambusinus* UFRN-Fungos 3222 (coll. AAL100); **(O)** *Mutinus caninus*; **(P)** *Xylophallus clavatus* INPA-Fungos 271637 (coll. TSC237). *Protophallaceae*: **(Q,R)** *Protubera maracuja* (coll. GCSM18). *Trappeaceae*: **(S)** *Restingomyces reticulatus* UFRN-Fungos 1890, holotype (coll. MAS335). Photographs: **(A)** Clark L. Ovrebo; **(B,E,F,L,O)** Manuel Tabarés; **(C,G,M,N)** Alexandro de A. de Lima; **(D,H,Q,R)** Gislaine C. S. Melanda; **(I)** Alexandre G. S. Silva-Filho; **(J,K,P)** Tiara S. Cabral; **(S)** Marcelo A. Sulzbacher.

On the other hand, the acceptance in *Phallales* of families composed of species characterized by sequestrate basidiomata (*Claustulaceae*, *Gastrosporiaceae*, *Gelopellaceae*, *Trappeaceae*, and *Protophallaceae*) has also generated controversies. For instance, morphological studies conducted by Zeller (1939, 1948) and Jülich (1981) considered *Gelopellaceae* and *Protophallaceae* in *Hysterangiales*, whereas Miller and Miller (1988) considered these families and *Claustulaceae* in *Phallales*. The last edition of Dictionary of the Fungi (Kirk et al., 2008) recognized three families in *Phallales*: *Claustulaceae* (=*Gelopellaceae*), *Gastrosporiaceae*, and *Phallaceae* (=*Clathraceae*; = *Lysuraceae*; = *Protophallaceae*), with *Trappeaceae* placed in *Hysterangiales*. Additionally, recent works based on *Basidiomycota* and general fungal classification (He et al., 2019; Wijayawardene et al., 2020) also considered *Trappeaceae* in *Hysterangiales* and only three families in *Phallales*: *Claustulaceae*, *Gastrosporiaceae*, and *Phallaceae* (Table 1). Representatives of some families of *Phallales* are shown in Figure 1.

There is still no consensus in family level systematics of *Phallales*, based on a compilation of sources (Table 1), such as works of fungal classification (He et al., 2019; Wijayawardene et al., 2020), taxonomic (Index Fungorum, 2020; MycoBank., 2020) and molecular databases (Nilsson et al., 2019; Schoch et al., 2020), and phylogenetic studies focused on *Phallales* (Hosaka et al., 2006; Degreef et al., 2013; Trierveiler-Pereira et al., 2014a; Sulzbacher et al., 2016). Thus, based on the importance of molecular data for systematics and phylogenetic studies and the fact that DNA databases can be a good tool to assess the history behind the sequences generated over the years, as well as the geographic distribution of certain taxa, we have undertaken this study using *Phallales* as a target group to retrieve sequences in molecular databases and to provide an overview of *Phallales* diversity represented by selected DNA markers available in public databases. Moreover, we aim to test the phylogenetic positioning of named and unnamed sequences; to assess phylogenetic hypotheses using combined markers to recognize families and genera and to compare these data with the extant classification; to recognize the total number of species hypothesis (SH) represented in *Phallales* based on internal transcribed spacer (ITS) sequence clustering; and, finally, to record the global geographic distribution of their representative genera, their lifestyle, and edibility.

## Materials and Methods

### Sequence Metadata

Our work used two databases to obtain *Phallales* sequences: NCBI GenBank^1^ and UNITE^2^. All sequences were downloaded on August 7 to 9, 2020, from both databases. GenBank is part of the International Nucleotide Sequence Database Collaboration and contains the vast majority of phalloid sequences from published articles. The UNITE database automatically clusters ITS sequences of eukaryotic organisms to approximately the species level (called SHs), and to facilitate unambiguous scientific communication, a DOI is given to each SH.

According to the revised bibliography already mentioned (Hibbett et al., 1997; Hosaka et al., 2006; Trierveiler-Pereira et al., 2014a; Sulzbacher et al., 2016; Kasuya et al., 2020), seven markers were selected to retrieve sequences in GenBank using query strings (Table 2): nuclear ribosomal ITS, nuclear large subunit rDNA (nuc-LSU), nuclear small subunit rDNA (nuc-SSU); mitochondrial small subunit rDNA (mt-SSU), mitochondrial protein-coding ATPase subunit 6 (*ATP*6), and nuclear protein-coding second largest subunit of RNA polymerase (*RPB*2); and nuclear protein-coding translation elongation factor subunit 1α (*TEF*1-α). GenBank query results were downloaded in TinySeq_XML format, and single datasets of nucleotide sequences were created for each marker. One single dataset of all ribosomal markers from GenBank was downloaded, and in order to identify each marker, this single dataset was separated manually, based on the marker name in each sequence title. From the UNITE database, the sequences under *Phallales* (DOI: TH005985) not placed in GenBank were retrieved manually. Furthermore, manual checking was done for some genera, as they are not classified in *Phallales* in GenBank (*Gastrosporium*, *Kjeldsenia*, *Phallobata*, *Phlebogaster*, and *Trappea*) and UNITE (*Phlebogaster* and *Trappea*).

**TABLE 2:** Query strings used to search for DNA sequences from phalloid fungi in GenBank.

Query	Query strings
Main query	txid68804[Organism] AND 300:10000[SLEN]
ITS; nuc-LSU; nuc-SSU; mt-SSU	rRNA[Title] OR ribosomal RNA[Title]
*ATP*6	ATP6[Title] OR ATPase6[Title] OR ATP synthase F0 subunit 6[Title] OR ATP synthase subunit 6[Title] OR MTATP synthase F0 subunit 6[Title] OR MT-ATP6[Title] OR MTATP6[Title] OR ATP-6[Title]
*RPB*2	rpb2[Title] OR rpbII[Title] OR RNA polymerase II second largest subunit[Title] OR RNA polymerase II second large subunit[Title] NOT rpb1
*TEF*1-α	TEF1[Title] OR EF1[Title] OR EF-1[Title] OR TEF-1[Title] OR TEF[Title] OR tef1a[Title] OR EF[Title] OR translation elongation factor 1[Title] OR EF1alpha[Title] OR EF1a[Title] OR EF1-alpha[Title] OR TEF1-alpha[Title]

Sequence metadata were retrieved from GenBank qualifiers and UNITE annotations. GenBank qualifiers include^3^ : *country*, *collection_date*, *culture_collection*, *environmental_sample*, *clone*, *isolate*, *isolation_source*, *lat_lon*, *organism*, *specimen_voucher*, *strain*, *tissue_type*, *type_material*, and *PCR_primers*, as well as information about authors, reference, title, and journal. UNITE annotations were obtained directly from the online database for each sequence; UNITE metadata include *sampling_area* (*country*), *sample_type* [Linked to (source)], and *collection_date*.

All ITS sequences obtained from GenBank and UNITE were used for additional Nucleotide BLAST searches in their respective database (NCBI BLAST, 2020; UNITE BLAST, 2020). These searches aimed to find ITS sequences of *Phallales* members that were deposited without being classified in this order. We retrieved unclassified sequences using the following cutoffs: query cover > 80%, identity > 70%, and e-value < e-1,000. The 80% sequence similarity represents the criterion to recognize the identity of sequences approximately at the order level (Tedersoo et al., 2014).

All information from the sequences retrieved from both databases and the BLAST searches was merged manually and organized in Supplementary Table 1 to better identify all markers of each individual based on their herbarium/culture accession number and/or other code given by whoever generated the sequences. An individual was considered repeated when it had more than one sequence of the same marker under different accession numbers or when it was indicated as a clone in the databases (individuals with “R” in the columns “R = repeated or duplicated voucher” of Supplementary Table 1). In these cases, two lines were created in Supplementary Table 1 for the same individual. The names of genera and species present in our Supplementary Table 1 are based on the qualifier *organism* in GenBank for each individual. In UNITE, the names adopted were according to the UNITE taxon name.

### Phylogenetic Positioning of Sequences and Recognition of Families and Genera

Phylogenetic analyses were performed using seven datasets, the combined one and the other six with each individual marker: ITS, nuc-LSU, mt-SSU, *ATP*6, *RPB*2, and *TEF*1-α. The marker nuc-SSU was not considered in the analyses because of the few (1%) sequences available (Supplementary Table 1). Sequences of *Hysterangiales* species were used as outgroup for individual marker analyses, as seen in Supplementary Table 2. The combined dataset (ITS + nuc-LSU + mt-SSU + *ATP*6 + *RPB*2 + *TEF*1-α) was constructed to confirm the organization in families, and this also used members of *Hysterangiales* as outgroup (Supplementary Table 3). Specimens from almost all genera retrieved in *Phallales* and that had most markers sequenced were chosen for the combined dataset (Supplementary Table 3), which includes 139 sequences: 69 ITS, 114 nuc-LSU, 26 mt-SSU, 76 *ATP*6, 65 *RPB*2, and 41 *TEF*1-α.

All datasets were aligned using MAFFT v.7 (Katoh and Standley, 2013) under the E-INS-i criteria. Seaview v.4 (Gouy et al., 2010) was used to visualize and adjust the alignments. The *RPB*2 alignment was partitioned into intron and exon, and *TEF*1-α alignment into intron 1/2/3 and exon 1/2 according to GenBank coding sequence notation. The best nucleotide substitution model was selected with BIC (Bayesian information criterion) using jModelTest 2v.1.6 (Darriba et al., 2012) for each individual dataset. Two strategies were used for phylogenetic reconstructions of each alignment: maximum likelihood and Bayesian inference. Maximum likelihood analyses were performed in RAxML v8.2.X (Stamatakis, 2006), combined with the rapid bootstrapping algorithm with 1,000 replicates under the GTRGAMMA option to obtain the maximum likelihood bootstrap (MLbs). Bayesian inferences were performed using MrBayes 3.2.6 (Ronquist et al., 2012) with two independent runs, each one beginning from random trees with four simultaneous independent chains, performing 2 × 10^7^ Markov chain Monte Carlo (MCMC) generations, sampling one tree every 1 × 10^3^ generation. The first 5 × 10^3^ sampled trees were discarded as burn-in, whereas the remaining ones (all sampled after the average standard deviation of split frequencies reached < 0.01) were used to reconstruct a 50% majority-rule consensus tree and to calculate Bayesian posterior probabilities (PP) of the clades. The jModelTest 2v.1.6, RAxML v8.2.X, and MrBayes 3.2.6 were run from the CIPRES Science Gateway 176 3.1 (Miller et al., 2010). All final alignments and the resulting topologies were deposited in TreeBASE under the number 28016.

### Species Hypotheses Recognition

The ITS sequences of *Phallales* retrieved were clustered using CD-HIT-EST (Huang et al., 2010) at 98.0% sequence similarity threshold (Tedersoo et al., 2014) to assess species hypotheses in *Phallales*.

### Geographic Distribution, Lifestyle, and Edibility

The qualifiers *country* and *lat_lon* from GenBank and *sampling_area* from UNITE were used to organize the global geographic distribution map of *Phallales* and each genus. To construct the maps based on this global distribution, the locality of each individual identified at genus level was retrieved from the databases (Supplementary Table 4). When possible, the missing localities in DNA databases were double searched in their original article or in secondary articles that give this information (Supplementary Table 4). When the geographic coordinate information was missing, we made an effort to establish it through Google Maps^4^ to complete the table. In the case of individuals for which the only origin information was the country, the geographic coordinates suggested by Google Maps were used based on the name of the respective country as a search keyword. One general map with all *Phallales* distribution was made, as well as separate maps according to genera.

To record the information related to lifestyle of each genus, we followed the FungalTraits database (Põlme et al., 2021) and additional data obtained from the qualifiers *environmental_sample* (GenBank), *isolation_source* (GenBank), and *sample_type* (UNITE). To complement and better explore the use of the phalloid species sampled with molecular data, the edibility status of the species was considered based on Li et al. (2021) and complementarily searched in the main text of the articles in which the sequences were generated.

## Results

### Sequence Metadata

**FIGURE 2:**
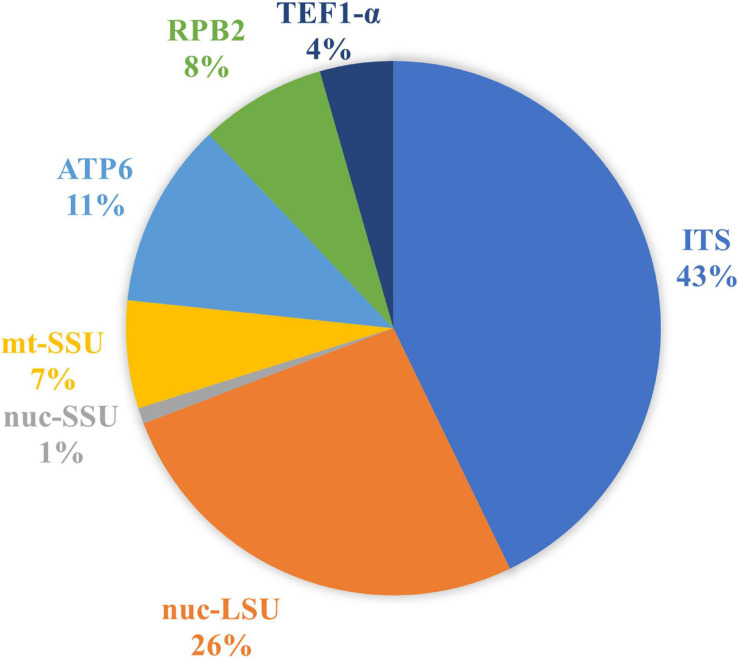
Relative proportion of phalloid DNA sequences deposited in DNA databases (GenBank and UNITE) according to the molecular marker.

Our final dataset led to a total of 1,149 DNA sequences of *Phallales* divided into 492 ITS, 303 nuc-LSU, 11 nuc-SSU, 75 mt-SSU, 129 *ATP*6, 88 *RPB*2, and 51 *TEF*1-α (Figure 2).

**FIGURE 3:**
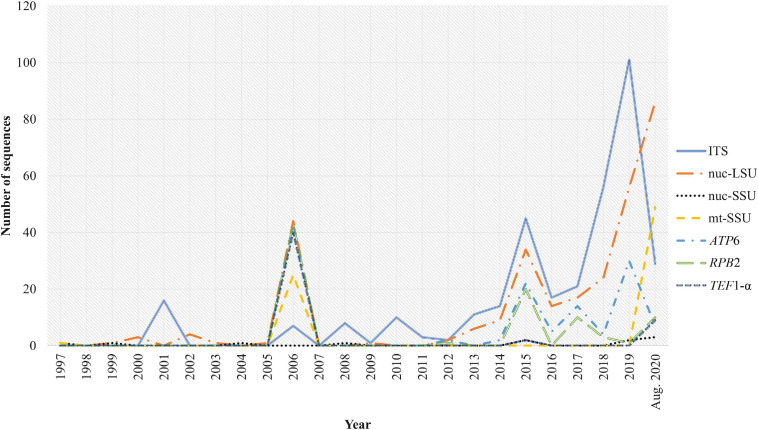
Number of *Phallales* DNA sequences uploaded in DNA databases (GenBank and UNITE) along the years.

Based on the available records of collection date (Figure 3), the first uploaded sequences of *Phallales* individuals were the nuc-SSU sequences of *Pseudocolus fusiformis* (E. Fisch.) Lloyd, dated October 31, 1997 (AF026623), and November 5 of the same year (AF026666), both part of the work published by Hibbett et al. (1997). In 2006, there was a peak of deposited sequences, including new markers such as *ATP*6, *RPB*2, and *TEF*1-α (Figure 3), and in 2012, there was a higher constancy of deposited sequences, in which ITS and nuc-LSU are the most represented markers (Figure 3). Interestingly, the sequence obtained from the oldest phalloid individual belongs to *Colus hirudinosus* Cavalier and Séchier (voucher UC 955042, dated February 1, 1952) and was uploaded in GenBank on April 4, 2020 (nuc-LSU accession code MK607412, author of sequence: Kuo, M.).

The 1,149 sequences comprise 664 individuals, with 19 of them comprising repeated sequences for the same maker. A total of 58.7% of all individuals have only one sequenced marker, 23.9% have two sequenced markers, and 17.4% have three to five sequenced markers. There are 122 sequences representing 41 type collections (Supplementary Table 5).

Sequences of 22 recognized genera were retrieved: *Abrachium* Baseia and T.S. Cabral, *Aseroë* Labill., *Blumenavia* Möller, *Clathrus* P. Micheli ex L., *Claustula*, *Colus* Cavalier and Séchier, *Gastrosporium*, *Gelopellis* Zeller, *Ileodictyon* Tul. and C. Tul., *Itajahya* Möller, *Kjeldsenia*, *Laternea* Turpin, *Lysurus* Fr., *Mutinus* Fr. (=*Jansia* Penz.), *Phallobata*, *Phallus* Junius ex L. (=*Dictyophora* Desv.), *Phlebogaster*, *Protubera* Möller, *Pseudocolus* Lloyd, *Restingomyces* (as *Phallales* sp.), *Trappea*, and *Xylophallus* (Schltdl.) E. Fisch. (Table 3). Sequences named *Gymnotelium* Syd. were retrieved as *Phallales*, but they are excluded from the present study because of their doubtful quality and because it is a genus classified in *Pucciniaceae* Chevall., *Pucciniales* Clem. and Shear (He et al., 2019). The genus *Calvarula* Zeller was not included in the combined analyses, because only one *TEF*1-α sequence is available and its placement at family level is questionable (see *Phylogenetic analyses*).

**TABLE 3:** Total of individuals and number of sequences of each molecular marker for each taxon retrieved from GenBank and UNITE databases from phalloid fungi searches.

Genus or name in sequence	N° unrepeated individuals	N° sequences	ITS	nuc-LSU	nuc-SSU	mt-SSU	*ATP*6	*RPB*2	*TEF*1-α
*Abrachium*	1	4	0	2	0	0	1	1	0
*Aseroë*	9	17	3	6	2	1	1	3	1
*Blumenavia*	17	51	11	12	0	1	8	10	9
*Calvarula* ?	1	1	0	0	0	0	0	0	1
*Clathrus*	42	83	24	34	0	6	9	8	2
*Claustula*	2	5	0	0	0	1	0	2	2
*Colus*	1	1	0	1	0	0	0	0	0
*“Dictyophora”*	43	52	41	3	0	2	2	2	2
*Gastrosporium*	6	14	7	7	0	0	0	0	0
*Gelopellis*	5	16	0	4	0	2	4	3	3
*Gymnotelium* ?	1	3	1	1	1	0	0	0	0
*Ileodictyon*	8	25	1	8	0	4	5	4	3
*Itajahya*	10	13	9	2	0	0	2	0	0
*“Jansia”*	14	17	14	1	0	0	1	1	0
*Kjeldsenia*	1	3	0	1	0	0	0	1	1
*Laternea*	1	5	0	1	0	1	1	1	1
*Lysurus*	38	98	17	31	0	12	20	16	2
*Mutinus*	59	89	37	31	1	9	7	4	0
*Phallobata*	1	4	0	1	0	0	1	1	1
*Phallus*	313	471	274	115	4	27	40	6	5
*Phlebogaster*	1	2	1	1	0	0	0	0	1
*Protubera*	28	88	6	22	0	6	21	20	13
*Pseudocolus*	8	15	5	7	1	2	0	0	0
*Trappea*	5	15	2	4	0	1	3	3	2
*Xylophallus*	3	4	0	3	0	0	0	1	0
*Basidiomycota* sp.	3	3	3	0	0	0	0	0	0
*Clathraceae* sp.	2	4	2	0	2	0	0	0	2
*Hysterangiales* sp.	1	1	1	0	0	0	0	0	0
*Phallaceae* sp.	3	3	1	2	0	0	0	0	0
*Phallales* sp.	2	3	2	1	0	0	0	0	0
*Phallales* sp. (*Restingomyces*)	2	5	1	2	0	0	2	0	0
uncultured *Agaricomycetes*	1	1	1	0	0	0	0	0	0
uncultured fungus	25	25	25	0	0	0	0	0	0
uncultured *Phallaceae*	3	2	3	0	0	0	0	0	0
uncultured *Pleosporales*	1	1	1	5	2	0	2	0	0
**Total**	**664**	**1109**	**492**	**303**	**11**	**75**	**129**	**88**	**55**

*Genus names followed by a question mark represent doubtful identification, and between quotes represent synonyms.*

*Phallus* is the most highly represented genus, with 313 individuals and 471 sequences (Table 3). Forty-three unidentified *Phallales* individuals were not classified at genus level (see individuals with “NO” in genus columns in Supplementary Table 1); they are specified in Table 3. The possible classification of these individuals is discussed in *Phylogenetic analyses*.

### Phylogenetic Analyses

The final aligned matrices for the analyses of each independent marker contain 481 sequences of ITS (1,210 positions), 306 nuc-LSU (1,245 positions), 77 mt-SSU (600 positions), 131 *ATP*6 (743 positions), 90 *RPB*2 (822 positions), and 53 *TEF*1-α (849 positions), and 139 of the combined matrix (4637 positions). The evolutionary models selected for the final dataset were as follows: ITS: TIM1 + I + G; nuc-LSU: TrN + I + G; mt-SSU: TPM3uf + G; *ATP*6: TVM + I + G; *RPB*2 Intron: TIM3 + I + G, *RPB*2 exon: TIM3 + I + G; *TEF*1-α intron 1: TrNef + G, *TEF*1-α exon 1: TrN + I + G, *TEF*1-α intron 2: TPM1 + G; *TEF*1-α exon 2: TPM1 + G, and *TEF*1-α intron 3: K80 + G.

**FIGURE 4:**
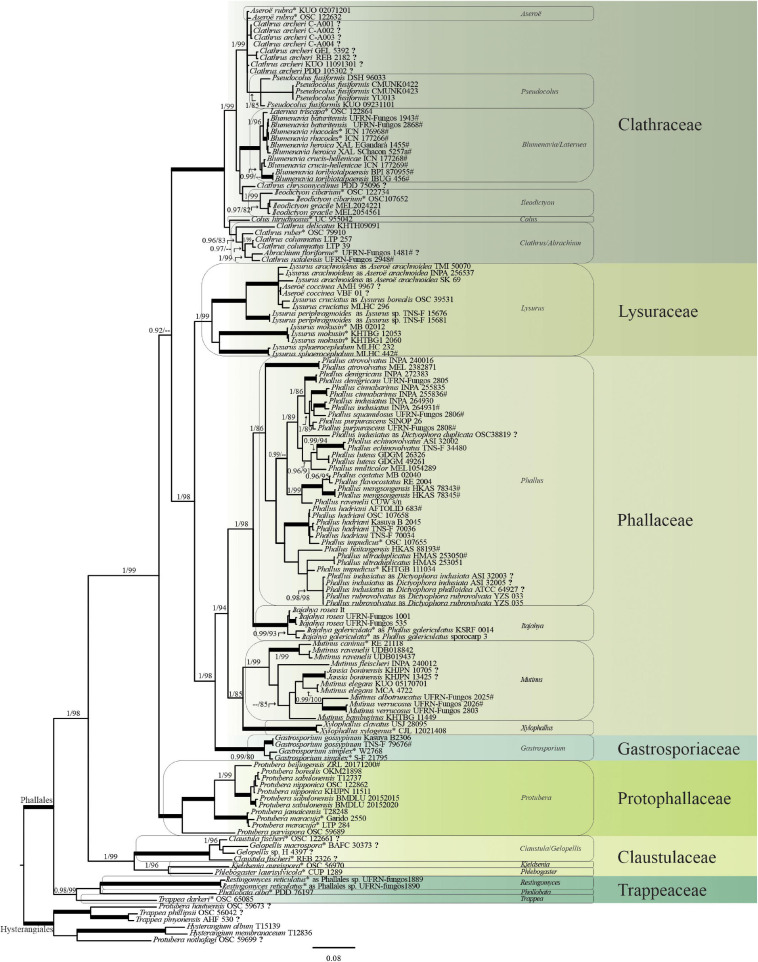
Phylogenetic tree of the *Phallales* based on a combined dataset (ITS/nuc-LSU/mt-SSU/*ATP*6/*RPB*2*/*TEF1-α). Family clades are colored in green shades and named on the right. Names of recognized genera are highlighted in the boxes. (*) Type species. (?) Sequences that need review of their identity or generic status. (#) Type specimens. Tree topology is based on the Bayesian analyses. Numbers on branches are posterior probabilities (PP, before slash) and maximum likelihood bootstrap values (MLbs, after slash). Thickened branches in boldface indicate fully supported nodes (PP = 1, MLbs = 100). Scale bar indicates expected changes per site.

The concatenated tree is shown in Figure 4, in which some names were changed according to the current name recognized by their phylogenetic positioning. The trees that resulted from the independent analyses of each marker are available in Supplementary Figures 1–3.

### Phylogenetic Positioning of Unnamed and Doubtfully Named Sequences

Two ITS sequences of environmental samples retrieved from GenBank (EF218792 and MF487330) did not match any phalloid sequence in BLAST, and thus, they were eliminated from our data and not included in Supplementary Tables 1, 3. Thirteen ITS sequences (MK518965, UDB015101 (JQ657782), MT644888, UDB018620, UDB0673787, UDB0317538, UDB0321542, UDB0215586, UDB0196057, UDB089976, MT512648, UDB0180761, and MH930315) and five nuc-LSU (MK518662, MH532563, MH532564, MH532565, and MH532566) were excluded from the dataset because they had many ambiguous bases and long gaps, possibly the result of poorly edited sequences. These sequences were also checked on NCBI BLAST, and they do not correspond to any species of *Phallales*. Six sequences were divided into ITS and nuc-LSU and incorporated in both ITS and nuc-LSU alignments (see individuals with “YES” in the column “nuc-LSU sequence incorporated in phylogenetic analyses…” in Supplementary Table 1).

Among the sequences that need revision because of possible misidentification or doubtful positioning are those of the monospecific genus *Calvarula*, as *Calvarula excavata* Zeller (*TEF*1-α, DQ219293), which is positioned in *Lysuraceae* (Supplementary Figure 1A), although its classification was in *Protophallaceae* (Zeller, 1939). Sequences named *Protubera* sp. (T20068), *Protubera hautuensis* Castellano and Beever (OSC59673), *Protubera nothofagi* Castellano and Beever (OSC59699), *Trappea phillipsii* (Harkn.) Castellano (OSC56042), and *Trappea pinyonensis* States (AHF530) are grouped outside the *Phallales* core, in the outgroup of *Hysterangiales*. Thus, these sequences, as well as *Hysterangium* sequences, were used to root the trees as representative of *Hysterangiales*, and their identification must be further investigated. Sequences named *Protubera* sp. (vouchers FLAS-F60616 and FLAS-F 61859), *Protubera canescens* G.W. Beaton and Malajczuk, and *Protubera clathroidea* Dring also need to be investigated because they clustered in *Clathraceae* or *Lysuraceae* (Supplementary Figures 1–3), despite the classification of the genus in *Protophallaceae* (Hosaka et al., 2006; Trierveiler-Pereira et al., 2014a,b). Finally, some sequences of *Gelopellis* need further studies because they clustered out of the expected *Claustulaceae* (Hosaka et al., 2006; Trierveiler-Pereira et al., 2014a); *Gelopellis purpurascens* G.W. Beaton and Malajczuk (voucher H292) is grouped in *Phallaceae* (Supplementary Figure 3B); *Gelopellis* sp. (voucher MEL 2063389) is in *Clathraceae* (Supplementary Figures 2B, 3A) or external to *Phallaceae* (Supplementary Figure 3B).

**FIGURE 5:**
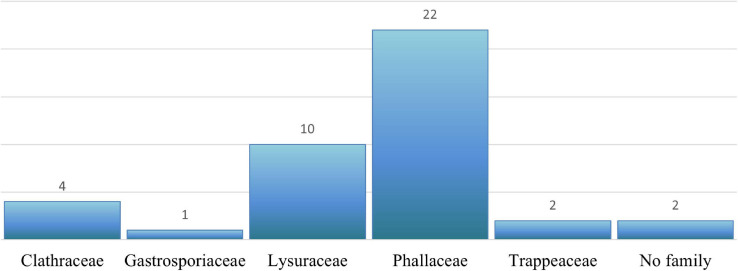
Number of undetermined *Phallales* individuals and their putative family level classification according to the phylogenetic position of the sequences recovered.

For the total of 43 unidentified individuals not classified at genus level (Table 3), 39 were placed in five distinct families, two did not group with any family in *Phallales* (Figure 5 and Supplementary Table 6), and the last two (*Hysterangiales* sp. UDB015101, and *Phallales* sp. UDB018620), as mentioned previously, were excluded from analyses. A total of 13 individuals were assigned to a specific genus: *Ileodictyon*, *Gastrosporium*, *Mutinus*, and *Phallus* (Supplementary Table 6). Fifteen individuals were identified to species level: *Blumenavia crucis-hellenicae* G. Coelho, Sulzbacher, Grebenc and Cortez, *Phallus impudicus* L., *Phallus hadriani* Vent., *Restingomyces reticulatus* Sulzbacher, B.T. Goto and Baseia (Supplementary Table 6). The identification of the sequences of *R. reticulatus* was possible by consulting the original article that proposed the new taxon (Sulzbacher et al., 2016). Supplementary Figures 1A, 2A,B, 3B show the phylogenetic positioning and the possible identification of some of the 41 unidentified individuals retrieved according to *TEF*1-α, ITS, nuc-LSU, and *ATP*6, respectively.

### Recognition of Families and Genera

Based on the phylogenetic inferences using the concatenated data matrix (ITS + nuc-LSU + mt-SSU + *ATP*6 + *RPB*2 + *TEF*1-α), our results show *Phallales* as a strongly supported monophyletic order (PP = 1, MLbs = 100) and composed of seven families (Figure 4). We recognized 22 genera in *Phallales*, with *Dictyophora* and *Jansia* confirmed as synonyms of *Phallus* and *Mutinus*, respectively, although the recognition of some genera and the placement of their representatives deserve attention: *Abrachium*, *Aseroë*, *Blumenavia*, *Clathrus*, *Claustula*, *Gelopellis*, *Laternea*, *Protubera*, *Pseudocolus*, and *Trappea*.

The composition of each family based on our analyses is presented below.

#### Clathraceae

This family grouped the genera *Abrachium*, *Aseroë*, *Blumenavia*, *Clathrus*, *Ileodictyon*, *Laternea*, and *Pseudocolus* (Figure 4). In the combined analyses, *Aseroë* in the *Clathraceae* clade is represented by the type species *Aseroë rubra* Labill. but is not recognized as monophyletic and forms a paraphyletic group with other sequences named *Clathrus archeri* (Berk.) Dring. These sequences warrant further examination. *Pseudocolus*, represented by *P. fusiformis*, is within the well-supported clade formed by *A. rubra* and *C. archeri*.

*Laternea*, based on the type species *Laternea triscapa* Turpin, forms a monophyletic clade together with sequences of *Blumenavia*, which includes the epitype specimen *Blumenavia rhacodes* Möller (voucher ICN 177266).

*Ileodictyon* is recognized as monophyletic (PP = 1, MLbs = 99), including seven sequences of two individuals of the type species *Ileodictyon cibarium* Tul. and C. Tul and another eight sequences of two individuals of *I. gracile* Berk. The specific identity of OSC107652, named as *I. cibarium*, must be investigated because it is positioned closer to sequences identified as *I. gracile*. Although with no support, *Clathrus chrysomycelinus* Möller is external to *Ileodictyon*, and the identity or generic status of the individual PDD75096 must be investigated.

*Colus* is represented by one nuc-LSU sequence of the type species *C. hirudinosus* Cavalier and Séchier, and it is closer to *Clathrus*, although this relationship is not supported.

*Clathrus* is recovered as polyphyletic with representatives clustered in at least three clades (Figure 4). Its core is represented by the type species *Clathrus ruber* P. Micheli ex Pers. plus *Clathrus delicatus* Berk. and Broome, *Clathrus columnatus* Bosc, *Clathrus natalensis* G.S. Medeiros, Melanda, T.S. Cabral, B.D.B Silva and Baseia, and *Abrachium floriforme* (Baseia and Calonge) Baseia and T.S. Cabral. *Abrachium* is represented here by only one collection (holotype of the type species). Sequences of *C. archeri* and *C. chrysomycelinus* are related to species of *Aseroë* and *Ileodictyon*, respectively; as mentioned previously, these should be further investigated for a possible reannotation or recombination.

#### Lysuraceae

In the family *Lysuraceae*, sequences named under *Lysurus* and *Aseroë* are included, although the latter has been formally classified in *Clathraceae*. Sequences are distributed in four well-supported clades (Figure 4). The most well-sampled clade includes sequences of *Lysurus arachnoideus* (E. Fischer) Trierv.-Per. and Hosaka (=*Aseroë aracnoidea* E. Fisch.), *Aseroë coccinea* Imazeki and Yoshimi, and *Lysurus borealis* (Burt) Henn. (=*Lysurus cruciatus* Henn.). Our results confirm the synonymizing of *A. aracnoidea* in *Lysurus* (Trierveiler-Pereira et al., 2014a), and based on the individuals retrieved, *L. borealis* is the confirmed synonym of *L. cruciatus* (Dring, 1980). The other three clades represent the following three taxa: *Lysurus periphragmoides* (Klotzsch) Dring from Japan (Caffot et al., 2018), the type species *Lysurus mokusin* (L.) Fr., and *Lysurus sphaerocephalum* (Schltdl.) Hern. Caff., Urcelay, Hosaka and L.S. Domínguez., with the latter considered an invalid name (Index Fungorum, 2020; MycoBank., 2020) according to Art. F.5.1 (Shenzhen), due to the absence of an identifier-issued citation in a recognized repository.

#### Phallaceae

This family is composed of *Itajahya*, *Phallus* (=*Dictyophora*), *Mutinus* (=*Jansia*), and *Xylophallus* (Figure 4). The type species *P. impudicus* is represented by two individuals that are positioned in two different clades, although the collection OSC107655 is closer to other sequences of *P. hadriani* Vent. and most likely is misidentified as *P. impudicus*. Anyway, the identity and positioning of *P. impudicus* deserve further investigations. Sequences of *Phallus indusiatus* Vent. closer to *Phallus rubrovolvatus* (M. Zang, D.G. Ji and X.X. Liu) Kreisel also most likely represent misidentification.

*Itajahya*, recognized as monophyletic and sister to *Phallus*, is represented by sequences of *Itajahya rosea* (Delile) E. Fisch. and *Itajahya galericulata* Möller. Sister to *Itajahya* and *Phallus* is the clade formed by *Mutinus* and *Xylophallus*. *Mutinus* is represented by eight species, including the type species *Mutinus caninus* (Huds.) Fr. and *Jansia boninensis* Lloyd. *Xylophallus* is represented by *Xylophallus clavatus* T.S. Cabral, M.P. Martín, C.R. Clement, K. Hosaka and Baseia and the type species *Xylophallus xylogenus* (Mont.) E. Fisch.

#### Gastrosporiaceae

*Gastrosporiaceae* is delimited in our analysis with high support (PP = 0.98, MLbs = 80) and sharing a common ancestor with *Phallaceae*, with both families (*Gastrosporiaceae* and *Phallaceae*) as sister of *Lysuraceae* (Figure 4). This monogeneric family is here represented by sequences of *Gastrosporium gossypinum* T. Kasuya, S. Hanawa and K. Hosaka, and the type species *Gastrosporium simplex* Mattir.

#### Protophallaceae

*Protophallaceae* is represented by the genus *Protubera* with the type species *Protubera maracuja* Möller plus *Protubera beijingensis* G.J. Li and R.L. Zhao, *Protubera borealis* S. Imai, *Protubera jamaicensis* (Murrill) Zeller, *Protubera nipponica* Kobayasi, *Protubera sabulonensis* Malloch, and *Protubera parvispora* Castellano and Beever (Figure 4). *Protubera parvispora* (OSC59689) was not placed in the *Protophallaceae* clade in the *ATP*6 tree (Supplementary Figure 3B), but it was in *Protophallaceae* in the other analyses (combined, nuc-LSU, *RPB*2, and *TEF*1-α).

#### Claustulaceae

In the *Claustulaceae*, clustered sequences were named *Claustula*, *Gelopellis*, *Kjeldsenia*, and *Phlebogaster* (Figure 4). *Claustula* and *Gelopellis* formed a monophyletic clade represented by four individuals including their type species: *Claustula fischeri* K.M. Curtis (type country New Zealand) and *Gelopellis macrospora* Zeller (type country Chile). *Gelopellis macrospora* is represented by a sample from Argentina, whereas *C. fischeri* is represented by two individuals from New Zealand, but these did not cluster together in our analyses, which puts into doubt the identification of one or more of these individuals as *C. fischeri*. The other clade in *Claustulaceae* is formed by *Kjeldsenia* and *Phlebogaster* represented by their type species: *Kjeldsenia aureispora* W. Colgan, Castellano and Bougher and *Phlebogaster laurisylvicola* Fogel, respectively. We retrieved two sequences (nuc-LSU and *TEF*1-α) of one individual of *P. laurisylvicola* (CUP 1289), which appears close to *Hysterangium* species in the nuc-LSU analyses (Supplementary Figure 2B) but is in *Claustulaceae* in the *TEF*1-α (Supplementary Figure 1A) and the concatenated analyses (Figure 4), which support our classification of *Phlebogaster* in *Claustulaceae* (*Phallales*).

#### Trappeaceae

This family is represented as monophyletic (PP = 0.98, MLbs = 99) in the combined analyses by *Restingomyces*, *Phallobata*, and *Trappea* with their respective type species *R. reticulatus*, *Phallobata alba* G. Cunn., and *Trappea darkeri* (Zeller) Castellano, respectively (Figure 4). However, the sequences of these genera were not recovered as a single-family clade in some of the unique marker analyses: in *TEF*1-α (Supplementary Figure 1A) and *RPB*2 (Supplementary Figure 3A), *P. alba* and *T. darkeri* are recovered separately; in ITS (Supplementary Figure 2A), *R. reticulatus* and *T. darkeri* do not form a clade (although the individuals of *T. darkeri* in the ITS analyses are not the same as all the other analyses); in nuc-LSU (Supplementary Figure 2B) *T. darkeri* is external (PP = 0.96) to all *Phallales*, whereas *R. reticulatus* and *P. alba* clustered together but with no support.

### Species Hypotheses Recognition

The clustering of the 479 sequences of ITS revealed 118 species hypotheses in *Phallales*, based on a sequence similarity threshold of 98.0%. In MycoBank. (2020), 576 legitimate specific names have already been deposited in *Phallales*. Our clustering shows that almost 20% of the total recognized species of *Phallales* have DNA sequences available, revealing that a lot of work remains to be done in this area.

### Notes on Geographic Distribution, Lifestyle, and Edibility

**FIGURE 6:**
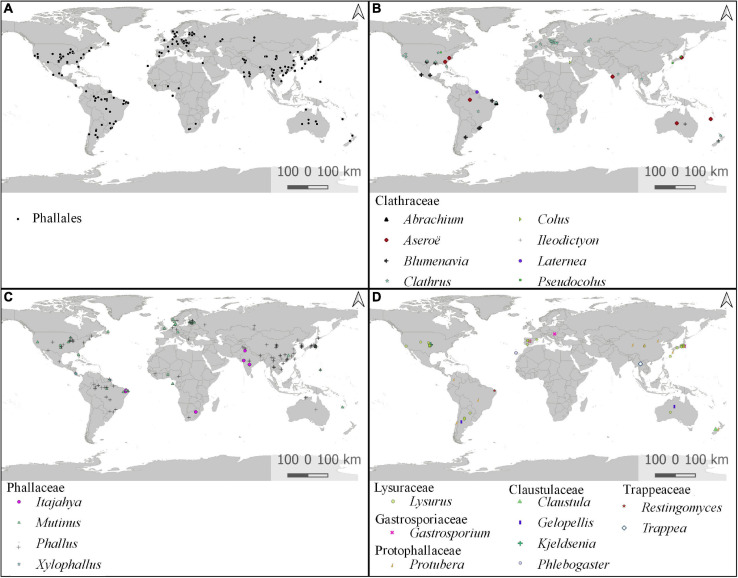
Global geographic distribution of *Phallales* based on samples with molecular sequences available and locality information. **(A)** Global distribution of all *Phallales* samples. Global distribution of **(B)** *Clathraceae* genera; **(C)** *Phallaceae* genera; **(D)** *Lysuraceae*, *Gastrosporiaceae*, *Protophallaceae*, *Claustulaceae*, and *Trappeaceae* genera.

Information on the country of origin included in DNA databases is available for 462 individuals (69.8%), from which 191 contain detailed information of the location but only 36 with the exact geographic coordinates. These individuals were distributed in 46 countries, which are concentrated in tropical and subtropical areas, with lower occurrence closer to the polar circles. Estonia is the source of the highest number of sequences (131) and individuals (131) deposited, all ITS. The United States is the second most sampled country, with 121 sequences of 79 individuals, and China is the third, with 108 sequences of 72 individuals. A map of global distribution of all phalloid individuals segregated by genera can be observed in Figure 6. For all 22 recognized genera, only *Phallobata* is not represented on the map, because there is no location information for the voucher. Individuals named under *Jansia* are represented as *Mutinus*, and those under *Dictyophora* as *Phallus* (Supplementary Table 4).

A total of 168 individuals (25.3%) are from environmental samples, including 148 individuals from soil, eight from roots of *Bouteloua gracilis* (Willd. ex Kunth) Lag. ex Griffiths (blue grama), two from air filters, three from seedling stem [two from *Hevea nitida* Mart. ex Müll. Arg and one from *Micrandra spruceana* (Baill.) R. E. Schult.], one from heavy metal–contaminated soil, one from marine subsurface sediments, and one from house dust. According to the FungalTraits database (Põlme et al., 2021), a total of 21 phalloid genera recognized in our work are soil saprotrophic, and only *Phlebogaster* is mentioned as ectomycorrhizal.

Among the *Phallales* sequences sampled in our work and their respective published references, only *Phallus dongsun* T.H. Li, T. Li, Chun Y. Deng, W.Q. Deng and Zhu L. Yang is reported by Li et al. (2020) as an edible species commercially cultivated in China. To complement the list of edible species of *Phallales* with molecular data available, according to edibility categories proposed Li et al. (2021), our study includes 11 confirmed edible species (E1): *Ileodictyon cibarium*, *Phallus echinovolvatus* (M. Zang, D.R. Zheng and Z.X. Hu) Kreisel, *Phallus fuscoechinovolvatus* T.H. Li, B. Song and T. Li, *P. hadriani*, *P. indusiatus*, *Phallus luteus* (Liou and L. Hwang) T. Kasuya, *Phallus merulinus* (Berk.) Cooke (as *Dictyophora merulina* Berk.), *P. rubrovolvatus*, *Phallus ultraduplicatus* X.D. Yu, W. Lv, S.X. Lv, Xu H. Chen and Qin Wang, *Protubera nipponica* [as *Kobayasia nipponica* (Kobayasi) S. Imai and A. Kawam.], and *Pseudocolus fusiformis*; three confirmed edible species, but with conditions (E2): *Clathrus archeri*, *Phallus impudicus*, and *Phallus multicolor* (Berk. and Broome) Cooke (as *Dictyophora multicolor* Berk. and Broome); six unconfirmed edible species (E3): *Clathrus columnatus*, *Ileodictyon gracile*, *Jansia boninensis*, *Lysurus mokusin*, *L. periphragmoides*, *Phallus rugulosus* (E. Fisch.) Lloyd; and three poisonous species (P): *Lysurus arachnoideus*, *Mutinus bambusinus* (Zoll.) E. Fisch., and *M. caninus*.

Molecular data are available for 81.5% of the *Phallales* species mentioned by Li et al. (2021) as having a known edibility status. Only *Mutinus borneensis* Ces., *Phallus armeniacus* Pat., *Phallus fragrans* M. Zang, and *Phallus tenuis* (E. Fisch.) Kuntze lack public sequence data. *Mutinus borneensis* and *P. armeniacus* are categorized by the authors as E3 (unconfirmed edible species), *P. fragrans* as E1 (confirmed edible species), and *P. tenuis* as P (poisonous species).

## Discussion

### Phallales Molecular Data From the Past 24 Years

The year 2006 can be marked as a huge advance in *Phallales* taxonomy with the work of Hosaka et al. (2006), a study aided by the Deep Hypha initiative (Blackwell et al., 2006). Hosaka et al. (2006) were responsible for 68 phalloid sequences deposited in GenBank (Figure 3), as well as the introduction of new markers (*ATP*6, *RPB*2, and *TEF*1-α). Another important turning point observed in Figure 3 is the increased use of molecular data in many works focused on *Phallales* diversity since 2013: nuc-LSU sequences – Degreef et al. (2013); ITS sequences – Moreno et al. (2013), Lu et al. (2014), Cabral et al. (2015), Kim et al. (2015), Elwess and Latourelle (2016), Patel et al. (2018), and Bobade and Dahanukar (2020); nuc-LSU and *ATP*6 sequences – Marincowitz et al. (2015); ITS and nuc-LSU sequences – Pietras et al. (2016); ITS, nuc-LSU, *RPB*1 and *ATP*6 sequences – Garnica et al. (2016). Trierveiler-Pereira et al. (2014a) also merit a spotlight because of their phylogenetic review of *Phallales* using nuc-LSU, *ATP*6, and *RPB*2 markers and by generating 53 sequences.

### Recognition of Families and Genera

#### Clathraceae

*Clathraceae* is characterized by fungi with clathrate (branched) or pseudostipitate and clathrate basidiomata that are named cage or lattice stinkhorns (Pegler and Gomez, 1994; Melanda et al., 2020). Individuals with clathrate format belong to *Blumenavia*, *Clathrus*, *Ileodictyon*, and *Laternea*, whereas those with prominent pseudostipe and a clathrate part composed of arms, armless, or lattice belong to *Abrachium*, *Aseroë*, *Colus*, and *Pseudocolus.* Cabral et al. (2012) accepted in *Clathraceae* the genera *Abrachium*, *Aseroë*, *Blumenavia*, *Clathrus*, *Pseudocolus*, *Lysurus*, and *Ileodictyon*. Our phylogenetic analyses, in agreement with Hosaka et al. (2006) and Trierveiler-Pereira et al. (2014a), show *Lysurus* as part of *Lysuraceae*. Based on morphological taxonomy using as the main diagnostic feature the disposition of the gleba in the receptacle, Pegler and Gomez (1994) classified the following genera in the *Clathraceae* series Lysuroid: *Aseroë*, *Colus*, *Lysurus*, *Kalchbrennera* Berk., *Neolysurus* O.K. Mill., *Pseudocolus*, and *Simblum* Klotzsch ex Hook. According to Pegler and Gomez (1994), these genera share the receptacle composed of a tubular and sterile pseudostipe, with the gleba attached to the upper portion of the receptacle. For Dring (1980), *Kalchbrennera* and *Simblum* were considered synonyms of *Lysurus.* However, our phylogeny (Figure 4) does not confirm this morphological approach as a natural character, with representatives of *Clathraceae* series Lysuroid sensu Pegler and Gomez (1994) in both *Clathraceae* (*Aseroë* and *Colus*) and *Lysuraceae* (*Lysurus*).

The relationship of the type species of *Aseroë*, *A. rubra* from Australia (type country), and *C. archeri* (Figure 4) has been previously shown by Hosaka et al. (2006), Degreef et al. (2013), and Trierveiler-Pereira et al. (2014a). The name *C. archeri* is a result of the combination of *Lysurus archeri* Berk. (Dring, 1980), which also has *Anthurus archeri* (Berk.) E. Fisch. as a synonym. The morphological research into *Clathraceae* performed by Dring (1980) considered *Anthurus* as a synonym of *Clathrus.* No other sequences of any other name previously treated as *Anthurus* are available. Considering that the type species of *Clathrus* is found in another clade, another generic name must be investigated for *C. archeri*, with *Anthurus* and *Aseroë* being two putative names, considering this taxonomic history and the relationship shown in our analyses.

The positions of *Laternea* and *Blumenavia* indicate that they may belong to the same genus, in which *Laternea* would have nomenclatural priority. *Blumenavia* and *Laternea* are also grouped in a monophyletic clade, as previously observed by Degreef et al. (2013) and Trierveiler-Pereira et al. (2014a), although both of these works performed their phylogenetic analyses with only one individual of each genus. Melanda et al. (2020) reviewed *Blumenavia* but did not present any *Laternea* species in the phylogeny. Considering that the molecular global sampling of *Laternea* is represented by only one representative, new molecular studies involving *Laternea* are encouraged, so as to better understand the relationship between *Laternea* and *Blumenavia*.

The genus *Colus* was considered as a member of the family *Clathraceae*, based on a morphological approach by Cunningham (1944), Dring (1980), and Pegler and Gomez (1994); here, we confirm this classification and also encourage the generation of more sequences of this genus.

*Clathrus* was shown to be polyphyletic by Hosaka et al. (2006) and Trierveiler-Pereira et al. (2014a). The relationship between *Abrachium* and *Clathrus* was observed by Trierveiler-Pereira et al. (2014a), who showed *A. floriforme* in an unsupported clade with *C. ruber* (type species) and *C. columnatus*. *Abrachium* is a monospecific genus proposed by Cabral et al. (2012), with the type species *A. floriforme* being a combination from *Aseroë floriformis* Baseia and Calonge. Cabral et al. (2012) emended the family *Clathraceae* to include the armless sunflower-shaped receptacle characteristic of *Abrachium*. Based on the high morphological variation between *Abrachium* and *Clathrus*, we have to wait for more sequences of abrachinoid individuals to infer any synonymy among the two genera, with *Clathrus* having nomenclatural priority. *Abrachium* appears to be widely distributed in the Atlantic Forest and Caatinga Biomes^5^, and additional studies may show unknown diversity.

*Linderia* G. Cunn, *Linderiella* G. Cunn., *Ligiella* J.A. Sáenz, and other possible representatives of *Clathraceae* do not have any sequences available and need to be included in further molecular phylogenetic studies. MycoBank. (2020) and Index Fungorum (2020) consider *Linderiella* and *Linderia* as synonyms of *Clathrus*, whereas He et al. (2019) consider only *Linderiella* as a synonym of *Clathrus*, but the inclusion of sequences of these genera in phylogenetic studies can confirm their relationships.

#### Lysuraceae

The positioning of the type species of *Lysurus* (*L. mokusin*, type country: China) from the United States and Japan has been previously shown by Trierveiler-Pereira et al. (2014a). *Simblum*, *Kalchbrennera*, and *Neolysurus*, other possible representatives of *Lysuraceae*, do not have any sequences available and need to be included in further studies. *Simblum* and *Kalchbrennera* are considered synonyms of *Lysurus* by Dring (1980), He et al. (2019), and in Index Fungorum (2020), whereas MycoBank. (2020) considers only *Kalchbrennera* to be a synonym of *Lysurus*, but the inclusion of sequences of these genera in phylogenetic studies can clarify their relationships. *Lysurus periphragmoides* and *L. sphaerocephalum* (nom. inval.) are names that have been previously placed in *Simblum*, and the phylogenetic placement of sequences under these names in our analyses could confirm the synonymizing between *Lysurus* and *Simblum*. *Simblum periphragmoides* Klotzsch and *S. sphaerocephalum* Schltdl. were considered as heterotypic synonyms by Dring (1980), who proposed the combination *L. periphragmoides*, but this synonymizing was not accepted by Caffot et al. (2018), and based on our analyses (Figure 4) it is also not confirmed. Considering the positioning of sequences named *A. coccinea* in the *Lysuraceae*, we encourage further studies to investigate the generic status of this taxon and the identity of the individuals under this name.

#### Phallaceae

Li et al. (2014, 2016) provided phylogenetic data to show that *Dictyophora* and *Phallus* should be treated as a single genus, *Phallus*. Cabral et al. (2019) described a high diversity in *Phallus indusiatus*, a species complex well-known for the presence of an indusium; however, *P. indusiatus* actually represents at least four phylogenetic species. Li et al. (2020) published the new species *P. dongsun*, which is not included in our concatenated tree, and mentioned that samples named *P. impudicus* from China represent *P. dongsun*, as *P. impudicus* is a species described from Europe (the type locality of *P. impudicus*). As previously mentioned, the identity of the sequences named *P. impudicus* recovered in our analyses needs further examination in order to obtain a robust view of the phylogenetic position of the species.

Kreisel (1996) accepted the genus *Itajahya* as *Phallus* subgenus *Itajahya*. In the same work, the type species of *Itajahya*, *I. galericulata*, originally described from South Brazil (Möller, 1895), was combined in *Phallus galericulatus* (Möller) Kreisel (Kreisel, 1996). However, based on our analyses (Figure 4), *I. galericulata* is clustered in a clade separate from *Phallus* and together with other *Itajahya* species, which does not justify the classification and the synonymizing proposed by Kreisel (1996). For *Mutinus* and *Jansia*, however, they are considered synonymous as previously pointed out by Crous et al. (2017). The positioning of *Xylophallus* as sister to *Mutinus* has been previously shown by Trierveiler-Pereira et al. (2014a) and Crous et al. (2018).

There are no molecular data for *Aporophallus* Möller, *Floccomutinus* Henn., and *Staheliomyces* E. Fisch., all genera also accepted in *Phallaceae* (Index Fungorum, 2020; MycoBank., 2020), although *Floccomutinus* is considered a synonym of *Mutinus* by He et al. (2019) and in MycoBank. (2020). *Aporophallus* and *Staheliomyces* are monospecific genera, and their revision will be highly relevant. Sequences of taxa dealt under *Floccomutinus* could be useful to better assess their relationships with *Mutinus*.

#### Gastrosporiaceae

The monogeneric family *Gastrosporiaceae* was confirmed as part of *Phallales* in our study and also by previous authors: Kirk et al. (2008), Trierveiler-Pereira et al. (2014a), He et al. (2019), Kasuya et al. (2020), and Wijayawardene et al. (2020). The genus *Gastrosporium* contains three described species: *G. simplex* (type species) from Italy, *G. asiaticum* Dörfelt and Bumžaa from Mongolia, and *G. gossypinum* from Japan (Honshu). The positioning of the family *Gastrosporiaceae* external to *Phallaceae* in our phylogeny (Figure 4) rejects the suggestion by Hosaka et al. (2006), who did not include *Gastrosporium* in their analyses and thus suggested that the ancestor of *Clathraceae*, *Phallaceae*, and *Lysuraceae* could be the point of transition from sequestrated to expanded basidiomata in *Phallales*.

#### Protophallaceae

*Protophallaceae* is represented in our analyses only by *Protubera* species, as also shown by Trierveiler-Pereira et al. (2014a, b). *Protophallaceae* was proposed by Zeller (1939) with species of *Calvarula*, *Protophallus* Murril, and *Protubera*. *Calvarula* is not included in our combined analyses (Figure 4), and the identity of the individual EF5-1 as *C. excavata* needs to be investigated. Additional individuals and sequences of *Calvarula* are needed to clarify its family level positioning. *Protophallus* is considered a synonym of *Protubera* (Malloch, 1989; He et al., 2019), and the positioning of *Protubera jamaicensis* (=*Protophallus jamaicensis* Murrill) in our analyses (Figure 4) confirms this synonymization.

Sanshi and Kawamura (1958) analyzed specimens of *Protubera* from Japan and proposed a new genus named *Kobayasia* S. Imai and A. Kawam. to accommodate *Protubera nipponica*. However, Hosaka et al. (2006) and Trierveiler-Pereira et al. (2014a, b) have shown sequences of *P. nipponica* within the clade formed by other *Protubera*, as also shown in our analyses (Figure 4), which does not justify *Kobayasia* as a separate genus and confirms its synonymization under *Protubera*. *Kobayasia kunmingica* M. Zang, K. Tao and X.X. Liu is the only other species described in *Kobayasia*, and its phylogenetic positioning should be investigated to better understand the relationship between *Protubera* and *Kobayasia*.

The relationship of *Protubera borealis* (voucher OKM21898) and *P. sabulonensis* has been shown by Hosaka et al. (2006) and Cabral et al. (2012). Sanshi and Kawamura (1958) considered *Protubera borealis* under the genus *Protuberella* S. Imai and A. Kawam. as *Protuberella borealis* (S. Imai) S. Imai and A. Kawam. However, the recognition of *Protuberella* as a separate genus was not accepted by Hosaka et al. (2006), Cabral et al. (2012), and Li et al. (2018), and it is also not confirmed here (Figure 4), although it is still considered as a distinct genus by He et al. (2019) and in Index Fungorum (2020).

Besides the sequences of *Protubera* clustered in *Phallales*, some individuals (T20068, OSC59673, and OSC59699) named under *Protubera* (including *P. nothofagi* and *P. hautuensis*) clustered in *Hysterangiales* (our outgroup), in agreement with Hosaka et al. (2006) and Trierveiler-Pereira et al. (2014b), who classified *P. nothofagi* in *Gallaceae* and *P. hautuensis* in *Phallogastraceae*. However, based on the positioning of the type species *P. maracuja* in *Phallales*, this must be the best classification for *Protubera* and *Protophallaceae*. The positioning of the individuals named *Protubera canescens* and *P. clathroidea* in *Clathraceae* and *Lysuraceae*, respectively (Supplementary Figures 1–3), has been shown also by Hosaka et al. (2006), Degreef et al. (2013), and Trierveiler-Pereira et al. (2014b). According to May et al. (2010), *P. canescens* is an egg (immature) form of *Ileodictyon*, and for Trierveiler-Pereira et al. (2014b), these *Protubera* species out of *Protophallaceae* may be egg forms of expanded phalloid taxa or misidentification.

#### Claustulaceae

In this family clade, the four genera *Claustula*, *Gelopellis*, *Kjeldsenia*, and *Phlebogaster* clustered together as in Hosaka et al. (2006). In Trierveiler-Pereira et al. (2014a), the family clade was represented only by *Gelopellis* and *Claustula*. Although *Claustula* and *Gelopellis* clustered together, it would be important to have sequences of nondoubtful identification and more representatives of both genera to confirm whether they could be considered synonyms, of which *Claustula* has nomenclatural priority. Individuals of *Gelopellis* clustered out of *Claustulaceae*, in *Clathraceae* (Supplementary Figures 2B, 3A), or external to *Phallaceae* (Supplementary Figure 3B), and they also need revision to confirm their identity or generic status, mainly because immature basidiomata can lead to misidentification of some expanded phalloids, as seen in individuals of *Protubera*.

#### Trappeaceae

The composition of *Trappeaceae* in our study by the three genera *Restingomyces*, *Phallobata*, and *Trappea* is in agreement with Sulzbacher et al. (2016). *Trappea* is the type genus of the family as established by Castellano (1990), in a study that mentioned that the genus represents a transition between *Clathraceae* and *Hysterangium*, and proposed the type species of *Trappea* based on *Hysterangium darkeri* Zeller. The positioning of *Trappea* in *Phallales* as a member of *Trappeaceae* is based on an individual of *T. darkeri* without information on the location, as also previously shown by Hosaka et al. (2006) and Sulzbacher et al. (2016). As in *Protubera*, some species of *Trappea* are also placed in *Hysterangiales*, but considering the positioning of its type species in *Phallales*, we confirm the classification of *Trappea* in *Phallales*. The positioning of some *Trappea* in *Hysterangiales* has also been observed by Hosaka et al. (2006) and Trierveiler-Pereira et al. (2014b), and both authors showed *T. phillipsii* and *T. pinyonensis* positioned in the *Phallogastraceae* (*Hysterangiales*) family. The identity or the generic status of these individuals of *Trappea* in *Hysterangiales* needs further revision.

### Distribution, Lifestyle, and Edibility

The DNA databases contain many sequences of *Phallales* representatives without precise information on the collection location or even without any metadata. In addition, the sequences have a low representation across continents, which makes it impossible to draw any robust inferences about the biogeographical distribution of families, genera, species, and clades. Thus, we encourage the inclusion of DNA sequences from representatives of *Phallales* from underexplored locations, such as Africa and maritime Southeast Asia, as well as the sequencing of collections already deposited in herbaria around the world and of taxa not yet sequenced. These further data will contribute to a better understanding of the evolutionary processes and distribution patterns in *Phallales*.

The explanation for the high number of sequences from Estonia is the increase of studies of ITS using environmental samples and the high number of these sequences deposited in UNITE (see Supplementary Table 1). Other locations with a large number of *Phallales* records and DNA sequences are partly due to the proximity of the research centers and specialists in the group.

Studies of environmental biodiversity or other ecological approaches including phalloid species started in 2003 (Bidartondo et al., 2003) and has continued in a few works (Sato et al., 2012; Kellner et al., 2014; Skaltsas et al., 2019; Vu et al., 2019).

In the FungalTraits database (Põlme et al., 2021), all their genera in *Phallales* are classified as saprotrophic. The genera *Phallobata*, *Phlebogaster*, *Restingomyces*, and *Trappea* are classified as part of *Hysterangiales*, but we recognize them in *Phallales*. *Phlebogaster* is considered an ectomycorrhizal genus in FungalTraits. Thus, considering *Phlebogaster* in *Phallales*, as supported by our analyses, the *Phallales* lifestyle must be expanded to include both saprotrophic and ectomycorrhizal genera. The ectomycorrhizal habit of *Phlebogaster* is most likely based on Fogel (1980), who described *P. laurisylvicola* as a hypogeous taxon under the plant species *Laurus azorica* (Seub.) Franco (*Lauraceae* Lindley). Kreisel (2001), in a checklist of gasteroid fungi, also mentioned *P. laurisylvicola* in association with *L. azorica.* However, we did not find any confirmation and description of these associations as an ectomycorrhizal symbiosis. We regard this species as putatively ectomycorrhizal, but further investigation is needed.

Hosaka et al. (2006) cited *Protubera canescens* as the only *Phallales* ectomycorrhizal, but according to May et al. (2010), this species is an immature form of *Ileodictyon*, as mentioned previously. In addition, Trierveiler-Pereira et al. (2014b) affirmed that all *Protubera* species are saprotrophic as reported in FungalTraits (Põlme et al., 2021). Therefore, we suggest further investigation to clarify the ectomycorrhizal association in this phalloid species.

Wild edible fungi are an important renewable natural resource in some regions, constituting important sources of income and nutrition. Despite their unpleasant odor, many representatives of *Phallales* are edible but are considered mushrooms with little culinary value. Stinkhorns are the most popular edible *Phallales* (Boa, 1988). The stinkhorn *Phallus* is the genus with the largest number of species with edible status (14: nine E1, two E2, two E3, and one P), followed by *Lysurus* (three E3 and one P) and *Mutinus* (one E3 and two P) that include only poisonous and unconfirmed edible species (Li et al., 2021). *Phallus* was reported by Li et al. (2021) as a genus with 43 described species, whereas He et al. (2019) reported 34 known species. Based on these numbers, the percentage of confirmed edible species in the genus represents 20.9 to 26.5%; however, the number of known species is probably underestimated because MycoBank. (2020) lists approximately 125 legitimate specific names for the genus. For *Phallales*, considering the 576 legitimate specific names recognized in MycoBank. (2020), the number of confirmed edible species in the order represents only 2.1%, whereas the number of poisonous species represents only 0.7%. The proportion of the number of confirmed edible and poisonous species in *Phallales* in relation to the 118 SHs that we found is 10.2 and 3.4%, respectively. This confirms that the edibility of *Phallales* species is poorly explored.

Many stinkhorns are consumed in the egg stage because of their tasty flavor (Phillips et al., 2018). In Germany and North America, the egg stage of *Phallus* is sold canned or fresh (Læssøe and Spooner, 1994). In China, *L. mokusin* and *P. rubrovolvatus* (as *Dictyophora rubrovolvata* M. Zang, D.G. Ji and X.X. Liu) are considered edible (Læssøe and Spooner, 1994). In addition, *P. indusiatus* and *P. dongsun* stand out in China for their flavor, where they are commercially cultivated (Boa, 1988; Li et al., 2020) and represent an important economic product. Despite the nutritional and commercial importance, the consumption of wild stinkhorns is not recommended unless their taxonomic affiliation is known with certainty because some of them are poisonous, such as species of *Lysurus* and *Mutinus*. Additionally, some species of the same genus are confirmed edible, whereas others are controversial, such as *P. tenuis*, which is considered poisonous (Li et al., 2021).

## Conclusion

This work presents a summary of studies using molecular tools in *Phallales*. In general, as in other groups of fungi, these tools clarify results of previous studies based on morphology; also, the use of combined markers has allowed a clearer delimitation and positioning of the families and genera. Although we recognized seven families and 22 genera in *Phallales*, an extra effort is needed in taxonomic studies of the genera *Abrachium*, *Aseroë*, *Blumenavia*, *Clathrus*, *Claustula*, *Gelopellis*, *Laternea*, *Protubera*, *Pseudocolus*, and *Trappea*, because some inconsistencies in species identification and positioning of their representatives should be clarified. It is also necessary to include sequences of the genera *Aporophallus* (*Phallaceae*), *Floccomutinus* (*Phallaceae*), *Kalchbrennera* (*Lysuraceae*), *Ligiella* (*Clathraceae*), *Linderia* (*Clathraceae*), *Linderiella* (*Clathraceae*), *Neolysurus* (*Lysuraceae*), and *Staheliomyces* (*Phallaceae*). The DNA databases are an excellent source of molecular data, both when searching for sequences and in helping to understand the evolution of traits shown in previous studies, but missing metadata can be a stalemate for that. Therefore, it is vital to fill in the records in DNA databases correctly and accurately.

Since the inclusion of molecular tools for systematics studies, considerable progress has been achieved in the taxonomic and evolutionary study of fungi. Sequences of different markers deposited in databases represent a valuable repository that is still poorly exploited. The data we utilized have been shown to be a good, no-cost tool to clarify taxonomic and systematics problems, test phylogenetic position of misidentified sequences, examine the geographic distribution of groups, explore the ecology and use of phalloid species, and to visualize where our knowledge gaps are. We therefore encourage all mycologists to conduct extensive reviews of molecular data available for their particular fungal taxa of expertise.

## Data Availability Statement

The datasets presented in this study can be found in online repositories. The names of the repository/repositories and accession number(s) can be found in the article/Supplementary Material.

## Author Contributions

GM and NM designed the project. ALe, GM, and NM extracted the data. GM organized the metadata. ALi, AS-F, ALe, GM, NA, and RF analyzed the data. ALi and GM performed quantitative analyses. AS-F performed BLAST and phylogenetic analyses. ALe performed species hypothesis recognition. GM, MM, AS-F, ALi, ALe, TC, NM, and RF wrote the draft. IB, MM, and NM supervised the project. All authors reviewed and approved the manuscript.

## Conflict of Interest

The authors declare that the research was conducted in the absence of any commercial or financial relationships that could be construed as a potential conflict of interest.

## Footnotes

1 http://www.ncbi.nlm.nih.gov/

2 https://unite.ut.ee/index.php

3 http://www.insdc.org/documents/feature_table.html#7.3

4 https://www.google.com.br/maps

5 https://www.gbif.org/occurrence/map?taxon_key=7812817

## Supplementary Material

Supplementary Figure 1 Phylogenetic trees of the *Phallales* order obtained with *TEF*1-α **(A)** and mt-SSU **(B)**. Names in blue and question marks indicate individuals with uncertain position or that represent a misidentification. Individuals retrieved without a genus name are represented in red with the possible species name based on our analyses given first. Family clades are colored in green shades and named on the right. Tree topology is based on the Bayesian analyses. Numbers on branches are posterior probabilities (PP, before slash) and maximum likelihood bootstrap values (MLbs, after slash). Thickened branches in boldface indicate fully supported nodes (PP = 1, MLbs = 100). Scale bar indicates expected changes per site.

Supplementary Figure 2 Phylogenetic trees of the *Phallales* order obtained with ITS **(A)** and nuc-LSU **(B)**. Names in blue and question marks indicate individuals with uncertain position or that represent a misidentification. Individuals retrieved without a genus name are represented in red with the possible species name based on our analyses given first. Family clades are colored in green shades and named on the right. Tree topology is based on the Bayesian analyses. Numbers on branches are posterior probabilities (PP, before slash) and maximum likelihood bootstrap values (MLbs, after slash). Thickened branches in boldface indicate fully supported nodes (PP = 1, MLbs = 100). Scale bar indicates expected changes per site.

Supplementary Figure 3 Phylogenetic tree of the *Phallales* order obtained with *RPB*2 **(A)** and *ATP*6 **(B)**. Names in blue and question marks indicate individuals with uncertain position or that represent a misidentification. Individuals retrieved without a genus name are represented in red with the possible species name based on our analyses given first. Family clades are colored in green shades and named on the right. Tree topology is based on the Bayesian analyses. Numbers on branches are posterior probabilities (PP, before slash) and maximum likelihood bootstrap values (MLbs, after slash). Thickened branches in boldface indicate fully supported nodes (PP = 1, MLbs = 100). Scale bar indicates expected changes per site.

Supplementary Table 1 General data compilation of all *Phallales* sequences recovered.

Supplementary Table 2 GenBank accession numbers for the outgroup used in phylogenetic analyses for individual markers.

Supplementary Table 3 Taxa, individuals, and accession numbers of sequences used in phylogenetic analyses from the combined dataset.

Supplementary Table 4 Taxa, individuals, and their respective geographic location used for the global distribution map.

Supplementary Table 5 Type collections and collections of reference (REF.) in *Phallales* with the respective GenBank accession numbers. When the status of the type is unknown, only “type” is indicated.

Supplementary Table 6 Putative identification for the undetermined *Phallales* individuals recovered.
