# Supplementary figures and images for: An Overview of 24 Years of Molecular Phylogenetic Studies in Phallales (Basidiomycota) With Notes on Systematics, Geographic Distribution, Lifestyle, and Edibility
Source: Front Microbiol. 2021 Jul 9;12:689374. doi: 10.3389/fmicb.2021.689374 (PMC8299787; doi:10.3389/fmicb.2021.689374)

A

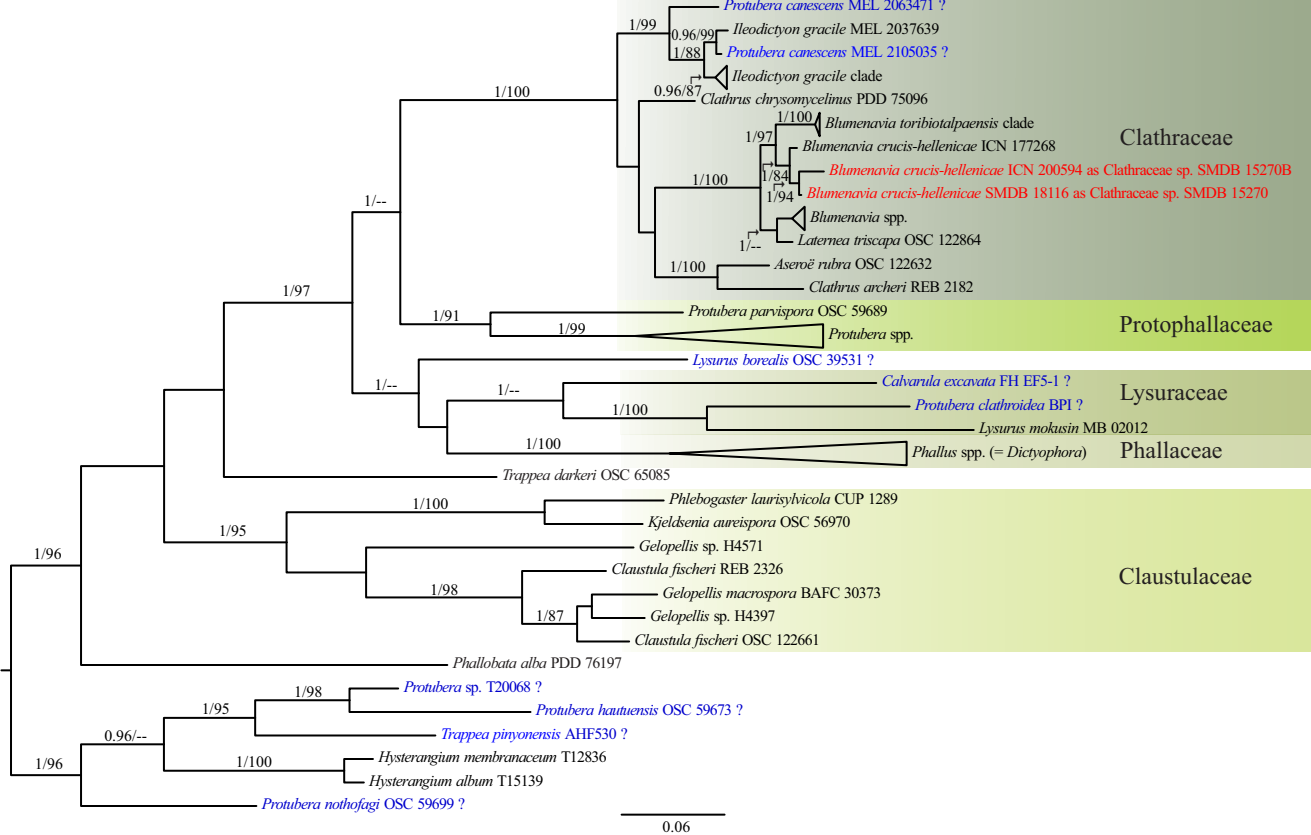

B

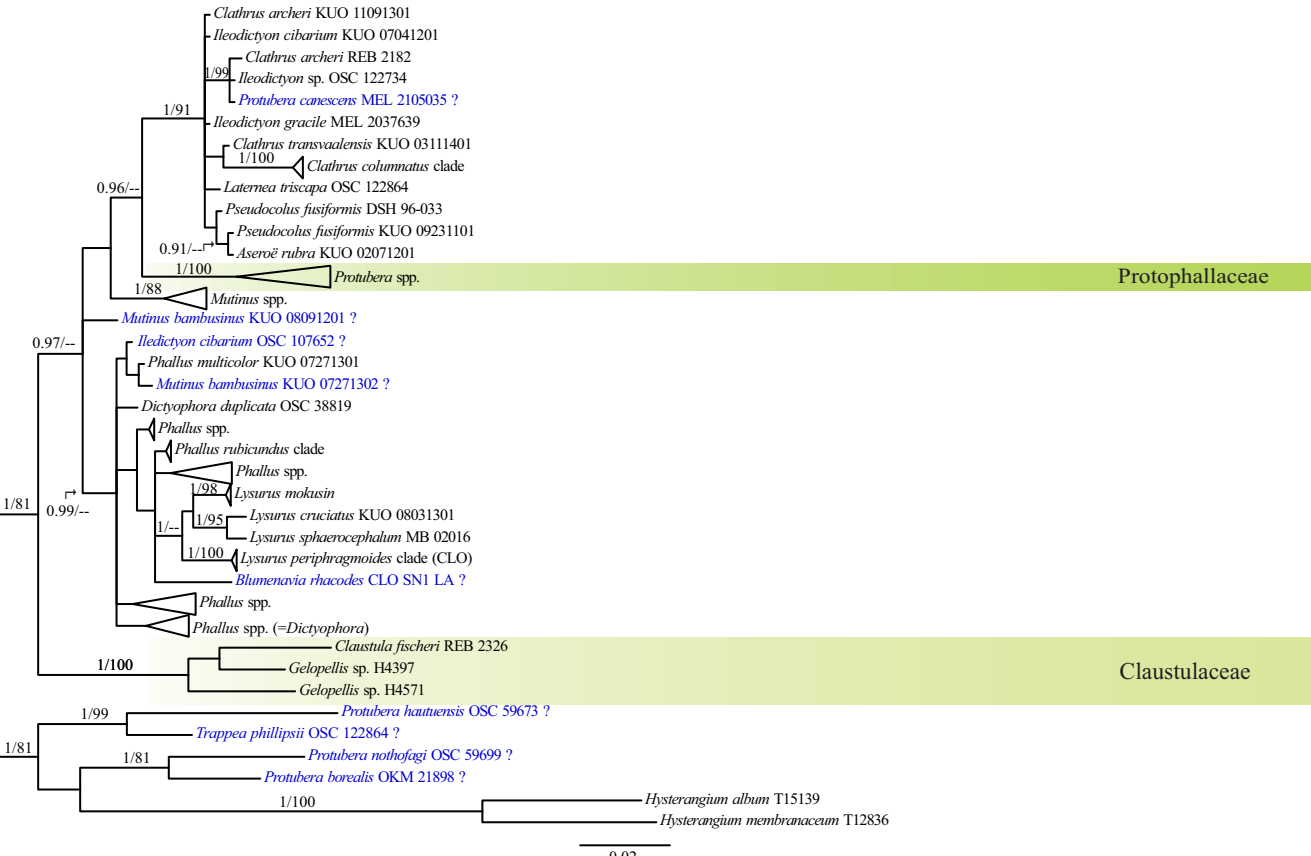

Supplement: Supplementary Figure 1 — Phylogenetic trees of the Phallales order obtained with TEF1-α (A) and mt-SSU (B). Names in blue and question marks indicate individuals with uncertain position or that represent a misidentification. Individuals retrieved without a genus name are represented in red with the possible species name based on our analyses given first. Family clades are colored in green shades and named on the right. Tree topology is based on the Bayesian analyses. Numbers on branches are posterior probabilities (PP, before slash) and maximum likelihood bootstrap values (MLbs, after slash). Thickened branches in boldface indicate fully supported nodes (PP = 1, MLbs = 100). Scale bar indicates expected changes per site. [file Image_1.pdf]

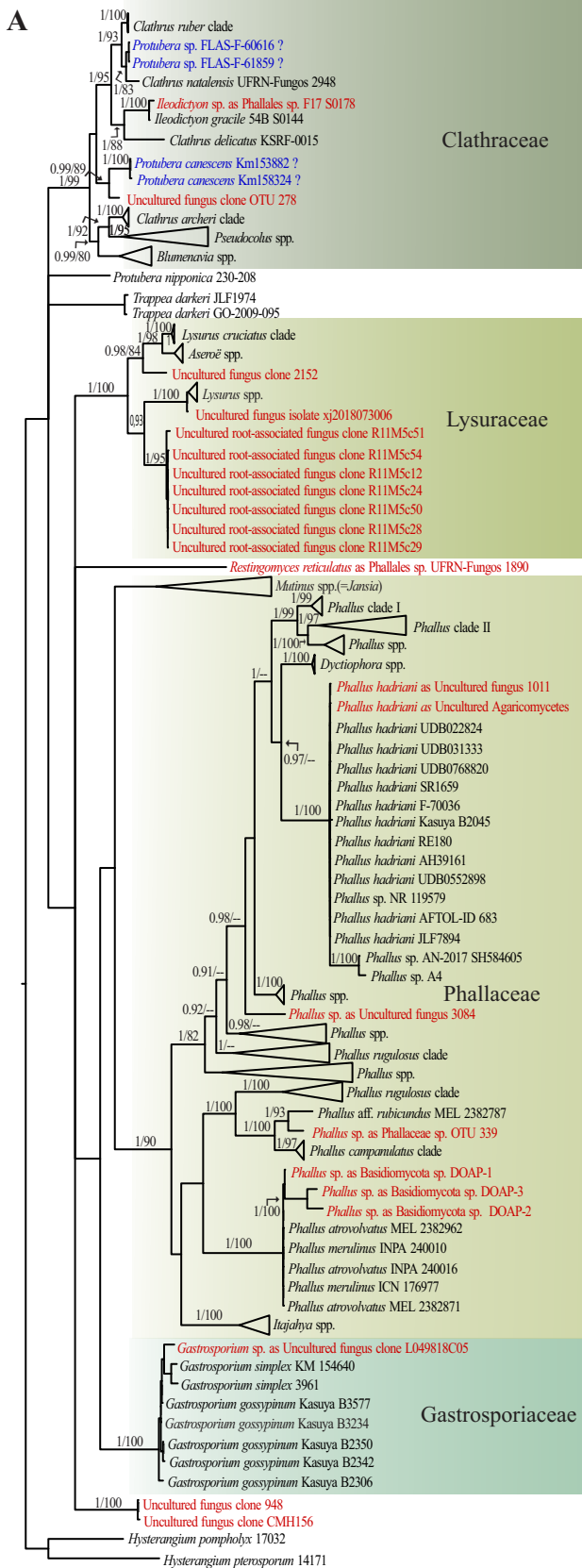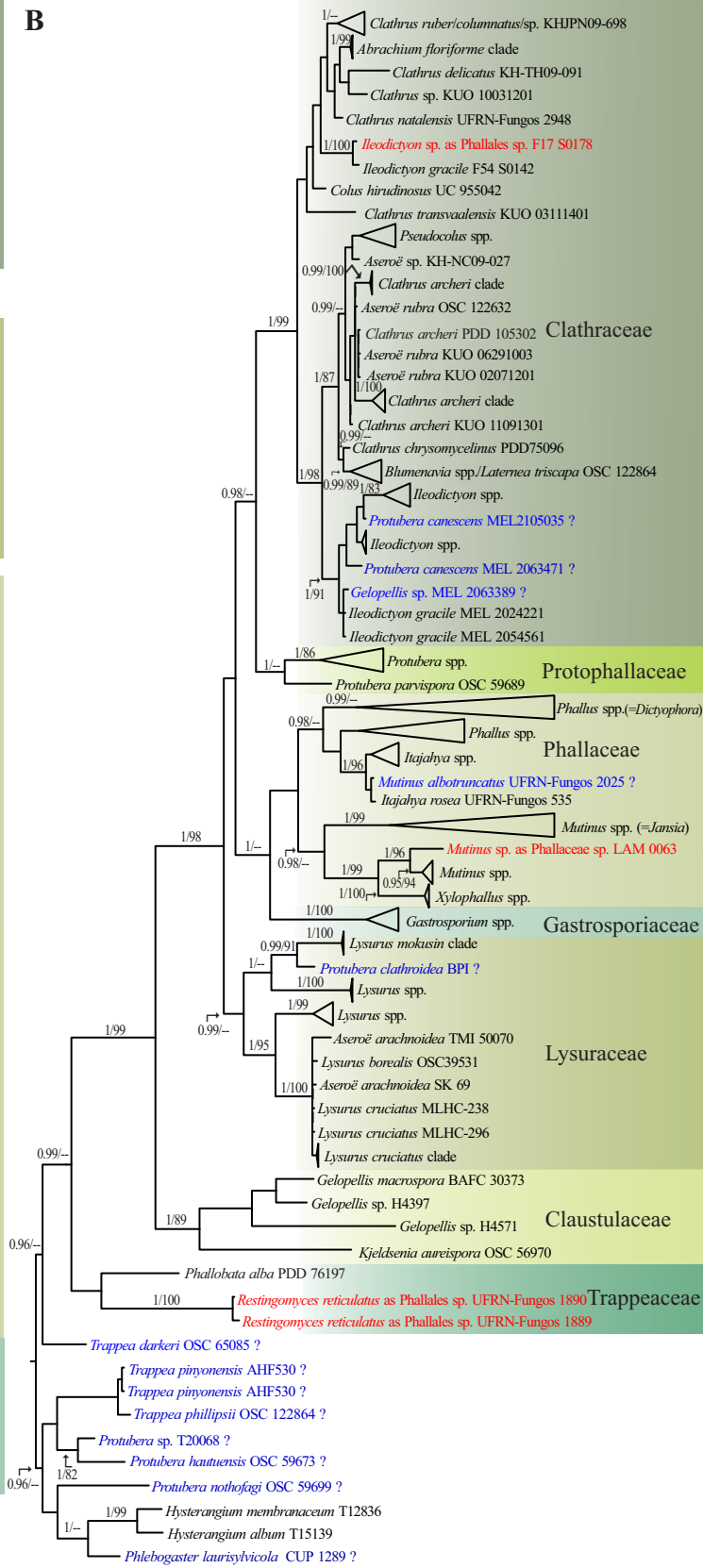

Supplement: Supplementary Figure 2 — Phylogenetic trees of the Phallales order obtained with ITS (A) and nuc-LSU (B). Names in blue and question marks indicate individuals with uncertain position or that represent a misidentification. Individuals retrieved without a genus name are represented in red with the possible species name based on our analyses given first. Family clades are colored in green shades and named on the right. Tree topology is based on the Bayesian analyses. Numbers on branches are posterior probabilities (PP, before slash) and maximum likelihood bootstrap values (MLbs, after slash). Thickened branches in boldface indicate fully supported nodes (PP = 1, MLbs = 100). Scale bar indicates expected changes per site. [file Image_2.pdf]

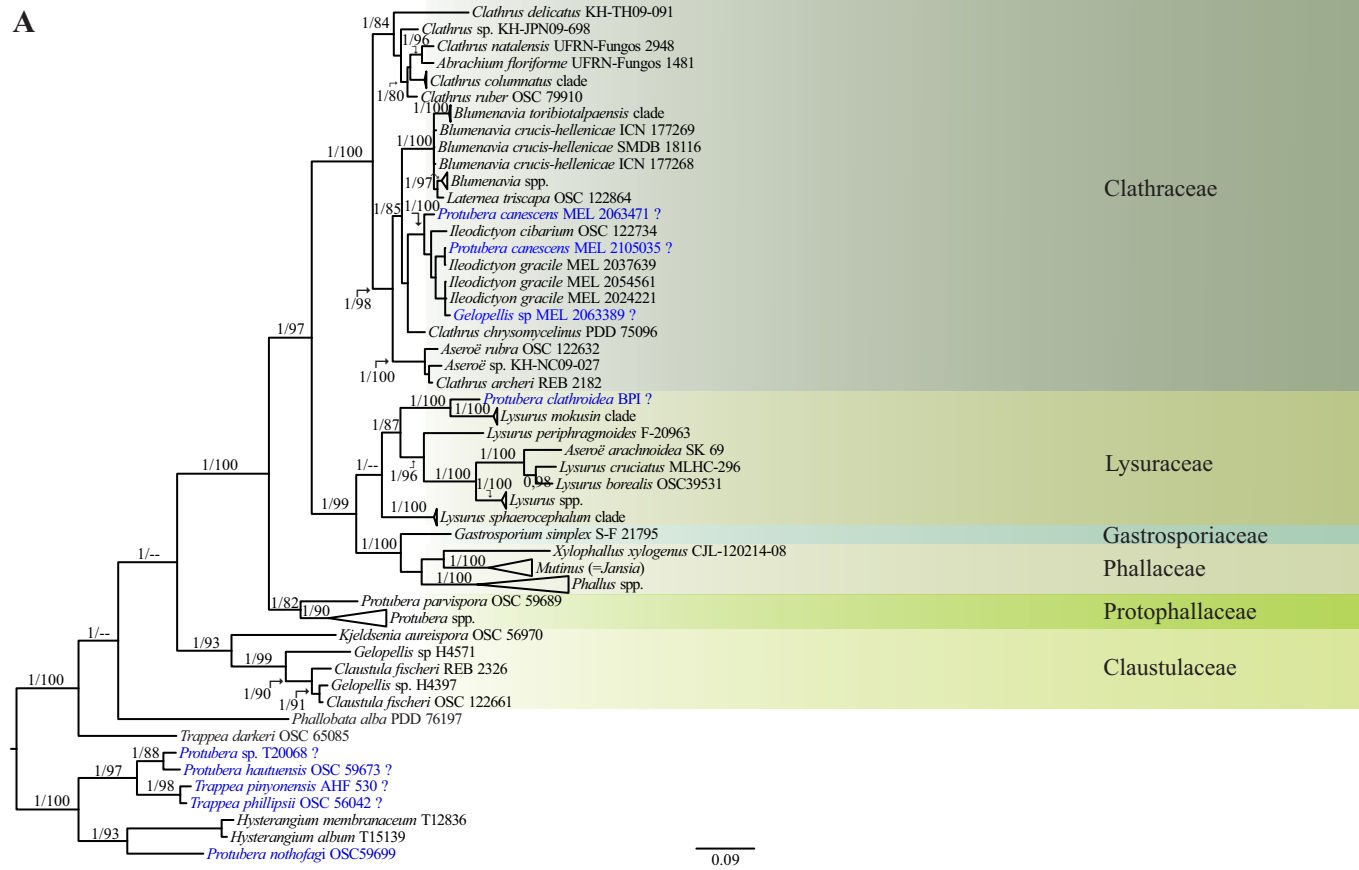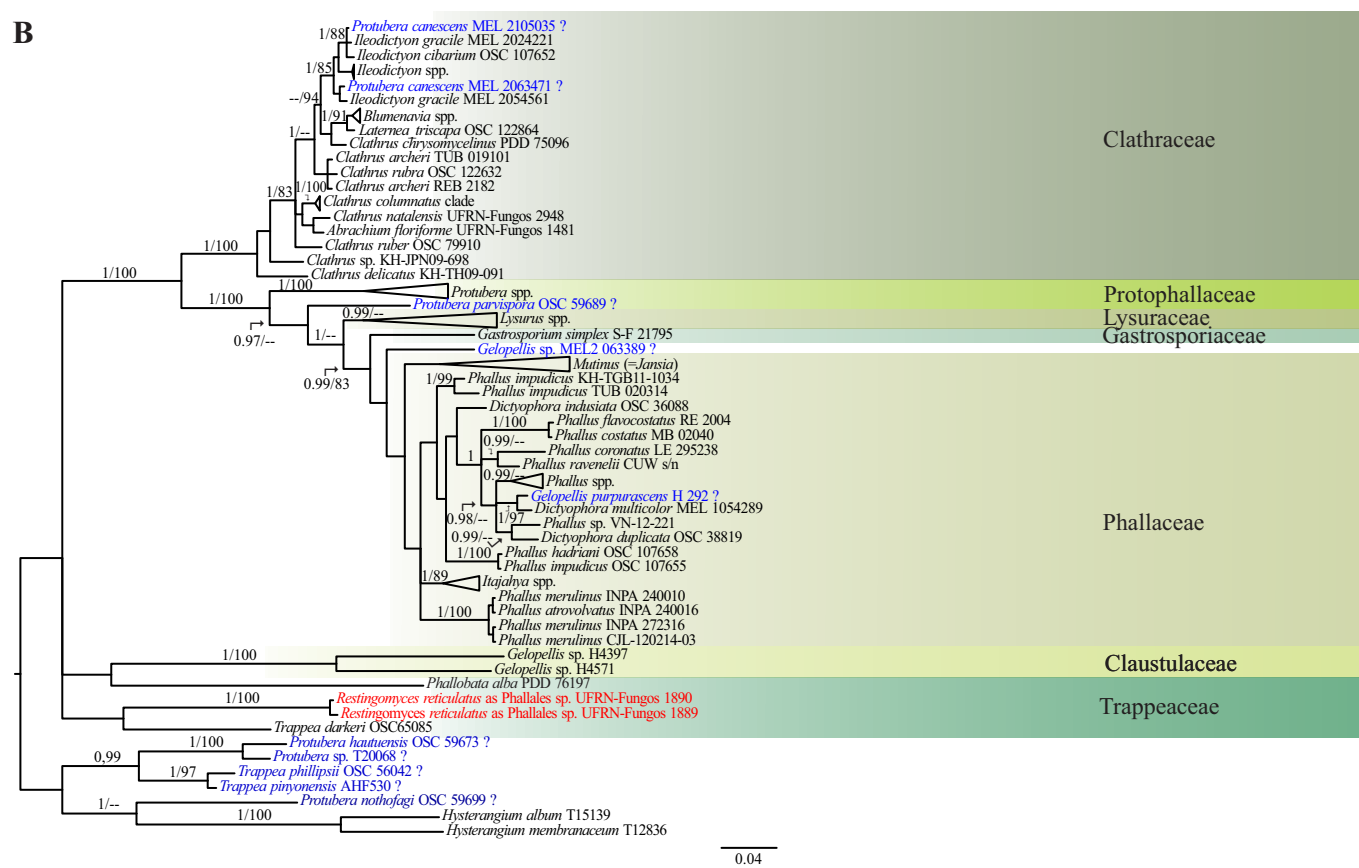

Supplement: Supplementary Figure 3 — Phylogenetic tree of the Phallales order obtained with RPB2 (A) and ATP6 (B). Names in blue and question marks indicate individuals with uncertain position or that represent a misidentification. Individuals retrieved without a genus name are represented in red with the possible species name based on our analyses given first. Family clades are colored in green shades and named on the right. Tree topology is based on the Bayesian analyses. Numbers on branches are posterior probabilities (PP, before slash) and maximum likelihood bootstrap values (MLbs, after slash). Thickened branches in boldface indicate fully supported nodes (PP = 1, MLbs = 100). Scale bar indicates expected changes per site. [file Image_3.pdf]
